# HiPSC-derived 3D neural models reveal neurodevelopmental pathomechanisms of the Cockayne Syndrome B

**DOI:** 10.1007/s00018-024-05406-w

**Published:** 2024-08-23

**Authors:** Julia Kapr, Ilka Scharkin, Haribaskar Ramachandran, Philipp Westhoff, Marius Pollet, Selina Dangeleit, Gabriele Brockerhoff, Andrea Rossi, Katharina Koch, Jean Krutmann, Ellen Fritsche

**Affiliations:** 1 IUF–Leibniz Research Institute for Environmental Medicine, Duesseldorf, Germany; 2 CEPLAS Metabolism and Metabolomics Laboratory, Cluster of Excellence on Plant Science (CEPLAS), Heinrich Heine University Duesseldorf, Duesseldorf, Germany; 3 Medical Faculty, Heinrich Heine University Duesseldorf, Duesseldorf, Germany; 4 DNTOX GmbH, Duesseldorf, Germany; 5 SCAHT, Swiss Centre for Applied Human Toxicology, University of Basel, Basel, Switzerland

**Keywords:** Disease modeling, In vitro, Personalized, Autophagy, Brain development, HDAC, MEA, Oligodendrocytes, GABA, Migration

## Abstract

Cockayne Syndrome B (CSB) is a hereditary multiorgan syndrome which—through largely unknown mechanisms—can affect the brain where it clinically presents with microcephaly, intellectual disability and demyelination. Using human induced pluripotent stem cell (hiPSC)-derived neural 3D models generated from CSB patient-derived and isogenic control lines, we here provide explanations for these three major neuropathological phenotypes. In our models, CSB deficiency is associated with (i) impaired cellular migration due to defective autophagy as an explanation for clinical microcephaly; (ii) altered neuronal network functionality and neurotransmitter GABA levels, which is suggestive of a disturbed GABA switch that likely impairs brain circuit formation and ultimately causes intellectual disability; and (iii) impaired oligodendrocyte maturation as a possible cause of the demyelination observed in children with CSB. Of note, the impaired migration and oligodendrocyte maturation could both be partially rescued by pharmacological HDAC inhibition.

**Graphical Abstract:**

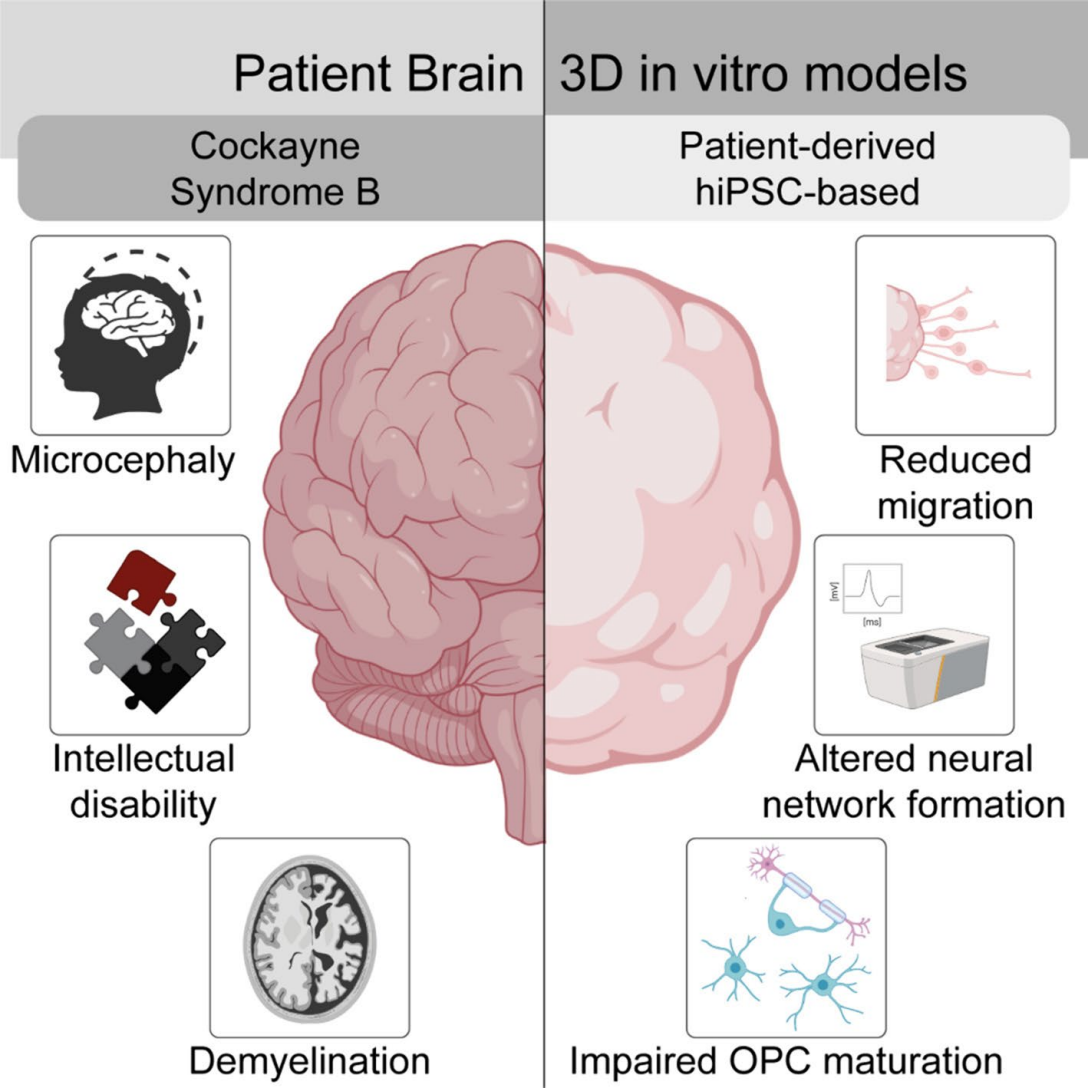

**Supplementary Information:**

The online version contains supplementary material available at 10.1007/s00018-024-05406-w.

## Introduction

The Cockayne Syndrome B (CSB) is a rare hereditary disease (prevalence **≈** 2.5 per million, [1]) with heterogeneous multi-organ defects including growth failure, retinal atrophy, deafness and a progeric skin phenotype. In addition, children with CSB develop severe neuropathological defects, with the cardinal phenotypes being microcephaly, intellectual disability and demyelination [2–6]. Around 70% of all CS cases are caused by several mutations in the excision repair 6 chromatin remodeling factor (ERCC) 6 gene and manifest with varying neurological severity, the most severe being fatal during early childhood [7, 8]. Some cases of CS have been linked to ERCC8 (CSA protein) defects, usually resulting in the less severe forms of the disease [9, 10].

CSB rodent models have proven very valuable for gaining insights into the clinical CSB phenotypes and the effects of CSB on the organism level [11–15], which lead to a good understanding of the mechanistic underpinning of the skin phenotype of CSB [16–18]. However, the origin of the children’s neurological defects is still enigmatic, because neurological defects cannot fully be modeled in rodents. Emerging in vitro approaches based on stem cells, e.g. human induced pluripotent stem cells (hiPSCs), can add to the current knowledge in human disease modeling and drug target identification, by providing excellent tools to investigate diseases and their underlying pathomechanisms [19–25].

CSB was originally found to be involved in the transcription-coupled nucleotide excision repair pathway (TC-NER) [26–29]. However, the neuropathology of the patients cannot be well explained with this mechanism. Previous in vitro studies have therefore suggested a role of CSB in the brain, which is independent of its involvement in the TC-NER [30–33]. Main findings include hindered neuronal differentiation and neuritogenesis, linked to reduced MAP2, as well as SYT9 and BDNF levels in 2D small hairpin (shRNA)-based CSB models of immortalized human neural progenitor cells (hNPC) and SH-SY5Y neuroblastoma cells [30, 31]. Vessoni et al. [32] found alterations in synapse density and reduced electrical activity in relation to a dysregulated Growth Hormone/Insulin-like Growth Factor-1 (GH/IGF-1) pathway in 2D iPSC-derived neuron/astrocyte mixed cultures and Liang et al. [33] identified Necdin as a CSB target, which promotes neuronal differentiation in 2D models of CS1AN human CSB fibroblasts and SH-SY5Y cells. Although these studies adumbrate the impact of CSB on brain development, we still lack the necessary understanding of why the clinical phenotypes arise and how we can treat them. Main limitations in current CSB models include non-physiological spatiotemporal microenvironments and low model complexity in 2D systems, missing cell types, such as oligodendrocytes, and a lack of appropriate control cell lines. These limitations outline the need for physiologically more relevant disease models, that better resemble the complexity of the developing human brain, and include isogenic controls. To investigate the brain phenotype of CSB in more physiologically relevant conditions, Szepanowski et al. [34] studied the effects of CSB on a transcriptomic level using 3D brain organoids derived from patient cells, providing valuable mechanistic information.

To add to this, we developed quality controlled CSB in vitro models, using patient derived and isogenic control cell lines, enabling the direct comparison between healthy and disease conditions and the identification of CSB-derived phenotypes. Study designs using multiple isogenic control pairs from different hiPSC donors have been shown to have an absolute power advantage of up to 60% compared to study designs without isogenic control pairs [35]. Here, we use such a favorable two isogenic pair design to study different neurodevelopmental processes disrupted in CSB. We closed the current knowledge gap on CSB neuropathology, by utilizing two hiPSC-derived fit-for-purpose 3D cell culture models, one of which is patient-based, including human induced neural progenitor cell (hiNPC) neurospheres and 3D-differentiated BrainSpheres [36–39]. The two different fit-for-purpose models were applied to answer different research questions concerning the underlying mechanisms of CSB. While hiNPC neurospheres enable the investigation of early developmental key events (KE) such as NPC proliferation, migration and initial terminal differentiation into neurons and astrocytes, modeling later KEs such as neural network formation and oligodendrogenesis benefit from more complex models such as 3D differentiated BrainSpheres [39]. BrainSpheres have a complex 3D cytoarchitecture and consist of the relevant brain cell types, i.e. neurons of different subtypes, astrocytes and—facultatively—oligodendrocytes. Together, both in vitro models serve as ideal tools for investigating earlier and later neurodevelopmental processes and the underlying mechanisms of their disruption [36–39].

## Materials and methods

### Cell lines

The commercial wild-type hiPSC-IMR90 line was obtained from WiCell (clone 4, Madison, USA). The patient-derived hiPSC-CS789 line was kindly provided by Prof. Egly from the IGBMC Strasbourg an has been sequenced at the IUF [40]. ERCC6 CRISPR/Cas9 mutants CS789^Res^ and IMR90^KO^ were generated in house, as previously described [41]. In brief, gRNAs (supplemental information (SI) Fig. S1) were designed using the CRISPR design tool CHOPCHOP (https://chopchop.cbu.uib.no/) and cloned into a modified version of the PX458 plasmid (Addgene #48138, Watertown, USA). The resulting bicistronic vector encoded the respective gRNA, Cas9 nuclease and GFP selection marker. gRNAs activity and efficiency were assessed via high resolution melt analysis (HRMA). HiPSCs cells were transfected with nuclease plasmids in antibiotic-free medium in a 6-well plate using Lipofectamine Stem (Thermo Fisher Scientific, Waltham, USA) or NEON electroporation system (Thermo Fisher Scientific, Waltham, USA). After 48 h, cells were sorted (FACS or MACS) and plated as single cells in a 96-well plate and duplicated after a week. Clones were lysed in proteinase K and genotyped by deep sequencing using a MiSeq Illumina (San Diego, CA) [41]. Briefly, libraries were quantified using qBit4 (Thermo Fisher Scientific, Waltham, USA) and deep sequencing was performed according to the manufacturer’s protocol (Illumina, San Diego, CA) at around 2000 reads per clone using custom made barcodes. Data were obtained in FASTQ format and analyzed using CRISPRnano.de [42].

To assure high and reliable cell culture quality, all hiPSC lines used in this study were quality controlled and banked, based on the recommendations of Tigges el al. [43]. Briefly, cells were characterized via karyotyping, STR analysis, FACS analysis for pluripotency markers and viability, mycoplasma test and colony morphology.

### Cell culture

The neural induction of all hiPSC lines and subsequent cultivation of hiNPCs in 3D was performed according to the Hofrichter et al. NIM protocol [36]. HiPSCs were maintained under feeder-free conditions on Matrigel-coated 6-well plates (LDEV-free, #354277, Corning, New York, USA) in mTeSR1 medium, containing mTeSR1 (#05850, StemCell Technologies, Vancouver, Canada), 20% (v/v) mTeSR1 supplement (5850, StemCell Technologies, Vancouver, Canada) and 1% (v/v) Penicillin/Streptomycin (P06-07100, PAN-Biotech, Aidenbach, Germany) for CS789, CS789^Res^ and IMR90^KO^, or on laminin-coated 6-well plates (#LN521-05, 50 µg/ml, Biolamina, Sundbyberg, Sweden) in iPSBrew iPSC medium, containing iPS-brew XF (human, 130-104-368, StemMACS, Miltenyi Biotec, Bergisch Gladbach, Germany), 2% (v/v) iPS Brew XF supplement (130-104-368, StemMACS, Miltenyi Biotec, Bergisch Gladbach, Germany) and 1% (v/v) Penicillin/Streptomycin for IMR90^WT^, at 37 °C in a humidified atmosphere of 5% CO_2_. This difference in cell culture media for the individual cell lines resulted from a change in the supply chain of media components. However, all cells were quality controlled extensively to ensure high quality and pluripotency throughout the experiment according to Tigges et al. [43]. Also, adequate cell performance was controlled to ensure similar behavior of the hiPSCs. The medium was changed on 6 days per week by completely removing and replacing the medium with fresh mTeSR1 or iPSBrew (2 ml) iPSC medium. On the sixth day of feeding, 4 ml of the respective medium were added, to substitute for the feeding-free seventh day. Passaging was performed with 0.5 mM EDTA (#15575020, Thermofisher Scientific, USA).

The neural induction of hiPSC cultures was initiated by incubating the cells with ROCK inhibitor (10 μM; ROCK INHIBITOR, #HB2297, Hello Bio, Great Britain) in mTeSR1 or iPSBrew medium for 1 h at 37 °C and 5% CO2. Subsequently, the cells were washed with PBS including 1% (v/v) Penicillin/Streptomycin (P06-07100, PAN-Biotech, Aidenbach, Germany) and neural induction medium (NIM; 1 ml), containing DMEM/F12 (31330038, Invitrogen, Waltham, USA), 1:50 B27 supplement (17504-044, Invitrogen, Waltham, USA), 1:100 (v/v) Penicillin/Streptomycin (P06-07100, PAN-Biotech, Aidenbach, Germany), 20 ng/ml Epidermal growth factor (EGF; PHG0313, Invitrogen, Waltham, USA), 20% (v/v) Knockout Serum Replacement (10828028, Invitrogen, Waltham, USA), 1:100 N2 supplement (17502-048, Invitrogen, Waltham, USA), 10 µM SB-431542 (S4317, Sigma Aldrich) and 0.5 µM LDN-193189 (SML0559, Sigma Aldrich, Burlington, USA) was added. Colonies were then fragmented with a StemPro EZPassage Disposable Stem Cell Passaging Tool (Thermo Fisher Scientific, Waltham, USA) and transferred into Poly-HEMA-coated 6 cm dishes (#P3932, Merck, Darmstadt, Germany) filled with NIM (5 ml). 10 µM ROCK inhibitor were added for at least 24 h. The medium was changed every second day. On day 7, spheres were collected and transferred into new Poly-HEMA-coated 6 cm dishes with 5 ml NIM and hFGF (10 ng/mL; #233-FB, R&D Systems, Minneapolis, USA) and the spheres were cultured for another 14 days. On day 21 the generated hiNPCs were transferred into new Poly-HEMA-coated 10 cm dishes filled with 20 ml NPC proliferation medium, containing DMEM/F12 (31330038, Invitrogen, Waltham, USA), 1:50 B27 supplement (17504-044, Invitrogen, Waltham, USA), 1:100 (v/v) Penicillin/Streptomycin (P06-07100, PAN-Biotech, Aidenbach, Germany), 20 ng/ml Epidermal growth factor (EGF; PHG0313, Invitrogen, Waltham, USA) and hFGF (20 ng/ml, #233-FB, R&D Systems, Minneapolis, USA). Cells were fed every second day with NPC proliferation medium and mechanically passaged to 0.2 mm diameter when the average sphere size exceeds a diameter of ≈ 0.5 mm, or when clumping occurs (McIlwain Tissue Chopper, Ted Pella, Redding, USA). Spheres were maintained in proliferation medium for subsequent cultivation. The hiNPC spheres were kept in culture and were regularly passaged when exceeding 0.5 mm in diameter. They were used for further experiments as early as passage 6 and no later than passage 20. All experiments were performed in biological replicates of 3, if not stated otherwise. These biological replicates were obtained from hiNPC spheres between passage 6 and 20, if not stated otherwise.

2D neural inductions were performed with cell lines IMR90^WT^ and IMR90^KO^, specifically for the generation of electrically active neural networks, which could not be achieved with the 3D induction protocol. The 2D inductions were performed according to Hartmann et al. [39]. HiPSC-colonies were dissociated using the Gentle Cell Dissociation Reagent (#100-0485, Stemcell Technologies, Vancouver, Canada) and subsequently seeded with a cell density of 2 × 10^6^ cells per well of a 6-well plate coated with polyethyleneimine (PEI, 0.1%; #181978, Sigma-Aldrich, Burlington, USA) and laminin (15 µg/ml; #LN521, Biolamina, Sweden), and cultivated in NIM medium supplemented with 10 µM ROCK inhibitor (only for the first 24 h after passaging; #HB2297, Hello Bio, Great Britain) under humidified conditions at 37 °C and 5% CO_2_. Cells were cultivated for 12 days, before medium was changed to neural progenitor medium (NPM), containing proliferation medium without hFGF, 20% (v/v) Knockout Serum Replacement (10828028, Invitrogen, Waltham, USA), 1:100 N2 supplement (17502-048, Invitrogen, Waltham, USA) and 20 ng/ml hFGF (#233-FB, R&D Systems, Minneapolis, USA). Medium was completely changed every second day. Cells were passaged on days 12 and 17 though enzymatic dissociation with Accutase (#07920, Stemcell Technologies, Canada) and transferred to a new PEI-laminin-coated 6-well plate. On day 21, hiNPCs were singularized with Accutase and frozen in neural progenitor medium containing 10% dimethyl sulfoxide (DMSO, #A994.1, Carl-Roth, Germany) and 10 µM ROCK inhibitor. Each thawn hiNPCs vial was diluted in 10 ml of the respective neural progenitor medium with 10 µM ROCK inhibitor (#HB2297, Hello Bio, Great Britain) and centrifuged at 300 × g for 5 min. The cell pellet was resuspended in 4 ml NPM medium with 10 µM ROCK inhibitor (#HB2297, Hello Bio, Great Britain) and transferred to one well of a 6-well plate (#83.3920, Sarstedt, Germany) coated with anti-adherence rinsing solution (#07010, Stemcell Technologies, Vancouver, Canada). Cells were cultivated in an orbital shaking incubator (#LT-X, Kuhner Shaker GmbH, Swiss) at 140 rpm, 12.5 mm diameter, 37 °C, 5% CO2, and 85% humidity for 7 days without feeding, to allow sphere formation. Medium was changed to NPC proliferation medium on day 7 for culture maintenance. Cells were fed every second day with NPC proliferation medium and mechanically passaged to 0.2 mm diameter when exceeding a size of ≈ 0.5 mm (McIlwain Tissue Chopper, Ted Pella). Spheres were maintained in proliferation medium for culture maintenance.

BrainSpheres differentiation was conducted according to the needs of the respective readout. Protocols are described in each section.

### Oligodendrocyte differentiation

Differentiation of hiNPCs from 3D neural inductions to oligodendrocyte-containing BrainSpheres was conducted based on the protocol published by Pamies et al. [44]. Proliferating hiNPC spheres were mechanically passaged to 0.1 mm diameter 1–2 days before the start of the differentiation. Spheres were transferred into 4 ml oligodendrocyte differentiation medium (ODM), containing Neurobasal Electro Medium (A1413701, Thermo Fisher, Waltham, USA), 1:50 B-27 Electrophysiology supplement (A1413701, Thermo Fisher, Waltham, USA), 1:100 Glutamax (A1286001, Thermo Fisher, Waltham, USA), 20 ng/ml human recombinant GDNF (212-GMP-010, RnD Systems, Minneapolis, USA), 20 ng/ml human recombinant BDNF (450-02, Peprotech, Rock Hill USA), 1% (v/v) Penicillin/Streptomycin (P06-07100, PAN-Biotech, Aidenbach, Germany), 1:100 db-cAMP (D0260, Sigma Aldrich, Burlington, USA), 60 ng/ml Triiodothyronine (T3; T2877, Merck, Darmstadt, Germany) and 20 µg/ml Ascorbic acid (A5960, Sigma Aldrich, Burlington, USA), per well of 6-well plates (#83.3920, Sarstedt, Germany) coated with anti-adherence rinsing solution (#07010, Stemcell Technologies, Vancouver, Canada). Spheres were cultivated free-floating, in an orbital shaking incubator (#LT-X, Kuhner Shaker GmbH, Swiss) at 140 rpm, 12.5 mm diameter, 37 °C, 5% CO_2_, and 85% humidity for up to 8 weeks. Half of the medium was changed for new ODM every second to third day. For rescue treatments between week 6 and week 8, medium was completely changed for ODM including 250 nM tubastatin A (#SML004, Sigma Aldrich, Missouri, USA) or 50 nM SAHA (#Cay10009929, Biomol, Hamburg, Germany). Exposed spheres were fed with ODM including the substance every second to third day until week 8.

### Migration

Cell migration was assessed as described previously, using hiNPC spheres neurally induced in 3D [45]. Briefly, one sphere of 0.3 mm diameter was plated per well of a 96-well plate coated with poly-d-lysine (PDL, 0.1 mg/mL, Merck, #P0899) and laminin (0.0125 mg/mL, Merck, #L2020). Spheres were cultivated under differentiation conditions in 100 µl CINDA medium, containing DMEM/F12 (31330038, Invitrogen, Waltham, USA), 1:50 B27 supplement (17504-044, Invitrogen, Waltham, USA), 1:100 N2 supplement (17502-048, Invitrogen, Waltham, USA), 644 mg/ml creatin monohydrate (C3630, Sigma Aldrich, Burlington, USA), 100 U/ml Interferon-γ (300-02, Peprotech, Rock Hill USA), 20 ng/ml Neurotrophin-3 (450-03, Peprotech, Rock Hill USA), 20 µM Ascorbic acid (A5960, Sigma Aldrich, Burlington, USA), 1:100 (v/v) Penicillin/Streptomycin (P06-07100, PAN-Biotech, Aidenbach, Germany) and 300 µM d-cAMP (D0260, Sigma Aldrich, Burlington, USA) for 3 days before the cells were imaged in brightfield mode (Cellomics ArrayScan, Thermo Fisher Scientific, Waltham, USA). Migration distance was measured from the sphere core to the furthest migrated cells using Fiji Image J software (v1.53f51, https://imagej.net/software/fiji/). Additionally, cells were fixed with 4% paraformaldehyde (#P6148, Sigma Aldrich, Missouri, USA) for 30 min at 37 °C, followed by three PBS washing steps. The nuclei were stained with 1% Hoechst (H21486, Thermo Scientific, Waltham, USA) and the cells were imaged (Cellomics ArrayScan). The Cellomics Spot Detection Tool was used to automatically count all cells outside of the sphere core, to assess the number of migrated nuclei. For treatment experiments, cells were cultivated in CINDA differentiation medium supplemented with SAHA (#Cay10009929, Biomol, Hamburg, Germany), Tubastatin A (#SML004, Sigma Aldrich, Missouri, USA), Chloroquine (Sigma, #C6628) or the respective solvent control. Endpoint assessment was performed as described above. Additionally, cell viability was measured with an Alamar Blue assay (Cell Titer-Blue® (CTB) Viability Assay; #G8080, Promega, Germany) according to manufacturer’s instructions. Cytotoxicity was assessed by measuring lactate dehydrogenase (LDH) exclustion (#G7890, CytoTox-One, Promega) following the manufacturer’s guidelines. Only non-cytotoxic concentrations were evaluated for statistical analyses, using a cut-off of 20% cytotoxicity.

### Proliferation

Human hiNPC spheres of 0.3 mm diameter were placed into separate wells of a Poly-HEMA-coated (#P3932, Merck, Darmstadt, Germany) U-bottom 96-well plate (Greiner, Austria) and statically cultured in proliferation medium for 3 days. The proliferation was assessed by measuring the increase in sphere size and by assessing the incorporation of BrdU into newly synthesized DNA. Spheres were imaged in brightfield mode (Thermo Fisher Scientific, Waltham, USA) from d0- to d3. The sphere size was automatically measured using the Cellomics ArrayScan Software and the slope of the size increase was calculated for each sphere. The cell proliferation BrdU assay was performed on day 3 (#11669915001, Sigma Aldrich, Missouri, USA) according to the manufacturer’s instructions.

### Immunocytochemistry (ICC)

Samples were fixed with a final concentration of 4% paraformaldehyde (#P6148, Sigma Aldrich, Missouri, USA) for 30 min at 37 °C, followed by three PBS washing steps. The desired primary antibodies were diluted in 2% goat serum (G9023, Sigma Aldrich, Missouri, USA) and PBS-T (PBS in 0.1% (v/v) Triton X-100 (#T8787, Sigma Aldrich, Missouri, USA)). Subsequently, the antibody solution was added to the samples and incubated at 4 °C overnight. Samples were washed three times with PBS. Next, the secondary antibodies, or conjugated antibodies, were added to 1% Hoechst (33258, Sigma Aldrich, Missouri, USA) and 2% goat serum in PBS. The samples were incubated with the secondary antibody solution at 37 °C for 1 h. Finally, the samples were washed three times with PBS and imaged with the confocal laser scanning microscope TCS SP8 (Inverse DMi8CS, Leica Microsystems) or the Cellomics ArrayScan (Thermo Fisher Scientific, Waltham, USA). Floating spheres were positioned onto microscopy glass slides and covered with Aqua-Poly/Mount (#18606-20, Polysciences Inc., USA) and a cover glass before imaging. All antibodies are listed in the supplementary table (SI Table S3).

ICC image detection and quantification by high-content imaging analysis (HCA) was done using the Cellomics ArrayScan. Respective channels were automatically acquired with a 40 × objective magnification and a resolution of 552 × 552 pixel. 30 randomly assigned fields of each 96-well were scanned. Automated image analysis was performed with the Thermo Scientific HCS Studio software, using the Colocalization analysis tool. Specifically, all O4 positive cells were detected and normalized to the total number of detected nuclei.

### RNA sequencing

IMR90^WT^ and IMR90^KO^ lines were used for transcriptome analyses. The cell lines IMR90WT/IMR90KO were chosen for RNASeq analyses due to their clean, non-patient genetic CSB knock-out ensuring prohibition of a potential bias through the patient genetic background and allowing a clean transcriptome analysis based on a truncated, non-functional CSB protein. Here, 500 hiNPC spheres with a 0.1 mm diameter were plated onto poly-d-lysine (PDL, 0.1 mg/mL, Merck, #P0899) and laminin (0.0125 mg/mL, Merck, #L2020)-coated 6-well plates and differentiated in CINDA medium for 3, 14 or 21 days. Total RNA was isolated using the RNeasy Mini Kit (#74104, Qiagen, Hilden, Germany) according to the manufacturer’s instructions. RNA was sent to BGI Genomics Co., Ltd. (China) for RNA sequencing using the DNBseq platform and the reads were mapped to human reference genome hg38. Three biological replicates were performed for each cell line.

Library preparation: Total RNA sample quality control (QC) was done using the Agilent 2100 Bio analyzer (Agilent RNA 6000 Nano Kit). Subsequently, mRNA was purified using oligo (dT)-attached magnetic beads, and fragmented. After, synthesis of the first and second cDNA strands, end repair and “A” base was added to the 3′end. Adaptor ligands were added, and PCR was performed. PCR product purification was done with XP beads. QC was again done using the Agilent 2100 Bio analyzer. Double stranded PCR products were denatured and circularized by splint oligo sequencing. Resulting single strand circle DNA was formatted as the final library. The library was amplified with phi29 to make DNA nanoball (DNB). The DNBs were loaded into the patterned nanoarray and single end 50 (pair end 100/150) bases reads were generated in the way of combinatorial Probe Anchor Synthesis (cPAS).

Sequence data analysis: First, reads mapping to rRNAs were removed. Next, low quality reads (> 40% of bases qualities < 20), reads with adaptors and reads with unknown bases (N bases > 0.1%) were removed, to get the clean reads (20 M clean reads per sample, BGI software SOAPnuke v1.5.2). These clean reads were stored as FASTQ files. Reads were subsequently mapped to the reference genome (GCF_000001405.39_GRCh38.p13) using the Hierarchical Indexing for Spliced Alignment of Transcripts software (HISAT2, v2.0.4). Additionally, novel transcript prediction (StringTie v1.0.4; Cuffcompare v2.2.1; CPC v0.9-r2), SNP & INDEL calling (GATK) and gene-splicing detection were done (rMATS v4.0.2). Gene expression analysis was performed, by mapping the clean reads to the reference genome (Bowtie2 v2.2.5) and calculating the expression levels with RSEM (v1.2.12). Differentially expressed genes (DEGs) between IMR90^WT^ and IMR90^KO^ lines were identified with DEseq2 [46].

### Quantitative polymerase chain reaction (qPCR)

For KCC2 and NKCC1 expression, 60 hiNPC spheres of 0.1 mm diameter were plated onto poly-d-lysine (PDL, 0.1 mg/mL, Merck, #P0899) and laminin (0.0125 mg/mL, Merck, #L2020)-coated 48-well plates and differentiated in CINDA for 3, 14 or 21 days. For oligodendrocyte marker expression analyses, spheres were differentiated as described in the oligodendrocyte differentiation protocol, before harvesting. Total RNA was isolated using the RNeasy Mini Kit (#74104, Qiagen, Hilden, Germany) according to manufacturer’s instructions. RNA was then reverse transcribed to cDNA (#205314, QuantiTec Reverse Transcription Kit, Qiagen, Hilden, Germany) according to the manufacturer’s instructions and qPCR was performed (#204057, Quanti Fast SYBR Green Kit, Qiagen, Hilden, Germany) using the PCR-Cycler Rotor-Gene Q (Qiagen, Hilden, Germany). Primer sequences are listed in the SI Table S2. Because we did not perform primer efficiency testing, the results have to be handled with care.

### Western blot (WB)

For CSB protein analyses, proliferating hiNPC spheres were analyzed. For all other markers, 750 hiNPC spheres with a diameter 0.1 mm diameter were plated into one well of a 6-well plate coated with poly-d-lysine (PDL, 0.1 mg/mL, Merck, #P0899) and laminin (0.0125 mg/mL, Merck, #L2020). Spheres were cultivated under differentiation conditions in CINDA medium for 3 days. Cell pellets of each well were lysed in RIPA buffer (Cell Signaling Technology, Massachusetts, USA) and 1 mM protease inhibitor PMSF (Cell Signaling Technology) on ice for 30 min and centrifuged for 15 min at 4 °C at maximum speed. Supernatant of protein samples were separated by 10% SDS-polyacrylamide gel electrophoresis and blotted onto PVDF membrane (BioRAD, Hercules, USA). Blots were blocked in 5% BSA diluted in 0.1% TBS-Tween-20 (TBS-T) for 1 h at RT and subsequently incubated with antibodies of interest overnight at 4 °C according to the manufacturer’s instructions. Blots were washed in TBS-T for 30 min and incubated with a 1:1000 dilution of HPR-conjugated secondary antibody (LI-COR Biosciences, Nebraska, USA) in 5% BSA in TBS-T at RT for 1 h. After final washing step of 30 min bands were visualized using ECL Prime (GE Healthcare, Freiburg, Germany) chemiluminescence substrate and the Odyssey imaging system (LI-COR Biosciences). Densitometry was carried out using Image Studio Lite software (LI-COR Biosciences). All antibodies are listed in the supplementary table (SI Table S3). All unprocessed blots can be found in supplementary figure S8.

### Multielectrode arrays (MEA)

96-well cyto-view MEA plates (#M768-tMEA-96B, Axion Biosystems, Atlanta, USA) were coated with poly-l ornithine (PLO, 0.1 mg/ml, #P3655, Sigma Aldrich, Missouri, USA) and laminin (#LN521-05, 50 µg/ml, Biolamina, Sundbyberg, Sweden). 3D induced hiNPCs of CS789^Res^ and CS789 cell lines were mechanically passaged to 0.1 mm diameter and transferred into CINDA+ differentiation medium, containing DMEM/F12 (31330038, Invitrogen, Waltham, USA), 1:50 B27 Plus supplement (A35828-01, Gibco, Billings, USA), 1:100 N2 supplement (17502-048, Invitrogen, Waltham, USA), 650 µg/ml creatin monohydrate (C3630, Sigma Aldrich, Burlington, USA), 100 U/ml Interferon-γ (300-02, Peprotech, Rock Hill USA), 20 ng/ml Neurotrophin-3 (450-03, Peprotech, Rock Hill USA), 20 µM Ascorbic acid (A5960, Sigma Aldrich, Burlington, USA), 1:100 (v/v) Penicillin/Streptomycin (P06-07100, PAN-Biotech, Aidenbach, Germany) and 300 µM d-cAMP (D0260, Sigma Aldrich, Burlington, USA). 20 hiNPC spheres in 200 µl CINDA+ were plated per well and 12 wells were prepared per cell line. For the IMR90^WT^ and IMR90^KO^ lines, 3D induced BrainSpheres did not yield sufficient electrical activity. Therefore, an adapted 2D induction protocol was used. Here, one proliferating sphere of approx. 0.2–0.3 mm size was plated in 200 µl CINDA+ per well and 12 wells were prepared per cell line. All lines were subsequently cultivated at 37 °C in a humidified atmosphere of 5% CO_2_ for up to 7 weeks. Cells were fed every two to three days, by removing 100 µl and adding 100 µl CINDA + medium. The electrical activity was measured for 15 min every week after an equilibration time of 15 min on the Maestro Pro MEA system (Axion Biosystems, Atlanta USA). During the time of the measurement, temperature and CO_2_ were kept stable and equivalent to the cultivation conditions. Data recording was operated by the Axion Integrated Studios (AxIS) navigator software (version 3.1.2, Axion Biosystems, Atlanta, USA) with a sampling frequency of 12.5 kHz and a digital band-pass filter of 200–3000 Hz. Subsequent spike detection was performed using the method “adaptive threshold crossing” with a threshold of 6 root mean square (rms) noise on each electrode and a pre- and post-spike duration of 0.84 ms and 2.16 ms, respectively. An electrode was termed “active” with at least 5 spikes per min. Quantification of general electrical activity and neuronal network activity was performed with the Neural Metric Tool software (version 3.1.7, Axion Biosystems, Atlanta, USA). For burst detection, the method “Inter-spike interval (ISI) threshold” was used with a minimum of 5 contributing spikes and a maximum ISI of 100 ms. Network bursts were identified using the algorithm “envelope” with a threshold factor of 1.5, a minimal inter-burst interval (IBI) of 100 ms, at least 35% participating electrodes, and 75% burst inclusion. Parameters for neuronal activity (percentage of active electrodes and number of spikes) as well as for network maturation and synchronicity (number of network bursts, number of spikes per network bursts and area under normalized cross correlation) were analyzed.

### GC-mass spectrometry

For GC–MS analyses, 500 hiNPC spheres with a diameter of 0.1 mm were plated into one well of a 6-well plate coated with poly-d-lysine (PDL, 0.1 mg/ml, Merck, #P0899) and laminin (0.0125 mg/ml, Merck, #L2020). Spheres were cultivated under differentiation conditions in CINDA medium for 14 days. As a wash control, 1 mM Tricarballylic acid (T53503, Sigma Aldrich, Missouri, USA) was added to each well of the cell culture medium, immediately before harvesting on day 14. Cells in each well were washed four times with ice cold 0.9% (w/v) saline in MilliQ water, before being collected in 2 ml of 0.9% (w/v) saline (3957.1, Roth, Karlsruhe, Germany) in MilliQ water. 2 ml methanol (N41.1, Roth, Karlsruhe, Germany) were supplemented with 250 µl internal standard (ISTD; 10 µM final; ribitol; A5502-5G, Sigma Aldrich, Missouri, USA). 2 ml methanol-ISTD solution was added to 2 ml of cell suspension and samples were shock frozen in liquid nitrogen. Upon thawing on ice, 1 ml chloroform (3313.1 Roth, Karlsruhe, Germany) was added to 4 ml sample solution and the mixture was incubated on ice and frequently vortexed for 10 min, before resting for 5 min on ice. The samples were centrifuged for 10 min at 4 °C and 4000 × g. Subsequently, the aqueous phase (top layer) was collected in a separate tube and the remaining organic phase was washed with 2 ml ice-cold MilliQ water. After another centrifugation cycle at 4 °C and 4000 × g for 10 min, the aqueous phase was collected and pooled with the first collection tube. The sample was filled with 11.5 ml MilliQ water, to reduce the amount of the organic solved < 15%. Samples were frozen for at − 80 °C, before lyophilization was carried out. Dried samples were resuspended in 500 µl MilliQ water, of which 20 µl were mixed with 50 µl methanol and dried via vacuum centrifugation for derivatization and GC–MS measurement.

The polar metabolites were derivatized for GC–MS analysis according to the method described by Gu et al. [47]. The derivatization process was executed using an MPS-Dual-head autosampler (Gerstel, Mülheim an der Ruhr, Germany). First, 10 µl of methoxyamine hydrochloride (10440364, Thermo Fisher Scientific, Fisher Scientific Chemicals, Waltham, Massachusetts, USA; freshly prepared at 20 mg/ml in pure pyridine from) were added, and the samples were shaken at 37 °C for 90 min. Next, 90 µl of *N*-Methyl-*N*-(trimethylsilyl)trifluoroacetamide (MSTFA; Macherey-Nagel, Düren, Germany) were added and shaken at 37 °C for 30 min, followed by a 2-h incubation at room temperature. Metabolite analysis was conducted using a 7890B gas chromatography system connected to a 7200 QTOF mass spectrometer from Agilent Technologies, as previously described by Shim et al. [48]. Compound identification was performed using MassHunter Qualitative software (v b08, Agilent Technologies, Santa Clara, USA) by comparing the mass spectra to an in-house library of authentic standards and the NIST14 Mass Spectral Library (available at https://www.nist.gov/srd/nist-standard-reference-database-1a-v14). Peak areas were integrated using MassHunter Quantitative software (v b08, Agilent Technologies, Santa Clara, USA) and normalized to the internal standard ribitol (Sigma Aldrich, Missouri, USA).

### Quantification and statistical analysis

#### Migration, proliferation, western blot and mass spectrometry analyses

GraphPad Prism was used to create visual graphs and analyses. Statistical significance was determined by unpaired two-tailed t-tests. Migration rescue experiments were analyzed using ANOVA with the Dunnett test for multiple comparisons. A p value below 0.05 was termed significant. Three biological replicates were performed for each cell line and each endpoint (three to four for WB). Migration and proliferation (including cytotoxicity and viability) additionally included three to ten technical replicates. Each biological replicate stems from a distinct neural differentiation.

#### qPCR

Fold change values were determined by 2^−∆∆Ct^. Statistical significance was determined by unpaired two-tailed t-tests in GraphPad Prism. A p value below 0.05 was termed significant. Three biological replicates were performed for each cell line. Each biological replicate stems from a distinct neural differentiation.

#### RNA seq

DEG criteria was set to |log2|≥ 1 and Qvalue of ≤ 0.05 were used for subsequent analyses and KEGG pathway mapping. Individually analyzed genes had a minimum FPKM (Fragments Per Kilobase per Million mapped fragments) of 1. Analyses and visualization of gene expression parameters were done using the Dr. Tom software (BGI Genomics Co., Ltd.). Three biological replicates were performed for each cell line. Each biological replicate stems from a distinct neural differentiation.

#### MEAs

Statistical analyses of MEA results were performed in GraphPad using Mixed-effects analysis with the Sidak test for multiple comparisons (N = 8–12 wells with n = 64–96 electrodes). Cells of one neural induction were used for all wells. A p value below 0.05 was termed significant.

## Materials and correspondence

Requests for further information should be directed to and will be fulfilled by the corresponding author, Dr. Katharina Koch (Katharina.Koch@IUF-Duesseldorf.de).

## Results

### Two hiPSC models for neurodevelopmental key event analyses in the cockayne syndrome B

**Fig. 1:**
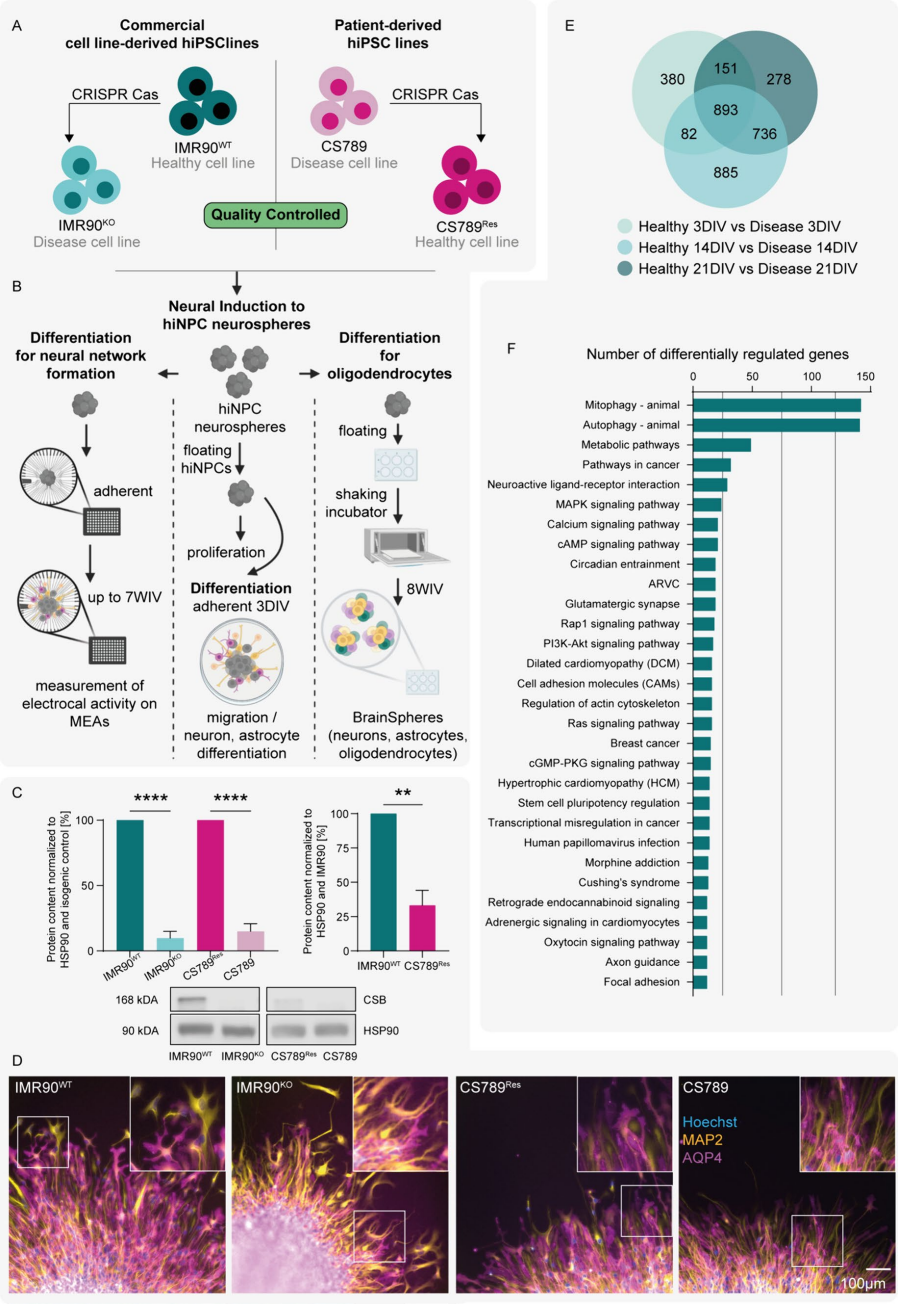
Human iPSC-derived cell models to study adversities in neurodevelopmental key events linked to Cockayne Syndrome B (CSB). **A** The two hiPSC-based CSB disease models used in this study include the commercially available IMR90^WT^ cell line and its CSB-deficient control line IMR90^KO^, as well as the patient-derived cell line CS789 and its isogenic control CS789^Res^, with partially restored CSB expression. Both IMR90^KO^ and CS789^Res^ were produced via CRISPR Cas (see also Supplemental Information Fig. S1). All hiPSC lines were quality controlled and cell banks were prepared according to Tigges et al. [43]. **B** Illustration depicting different fit-for-purpose models. Human iPSCs were neurally induced to hiNPC neurospheres, which were used to assess NPC proliferation after. HiNPCs were plated onto coated surfaces for subsequent differentiation, to assess NPC migration and differentiated into neurons and astrocytes after 3 DIV (middle). In order to obtain electrically active neural networks, hiNPC neurospheres were plated onto coated MEA plates for subsequent differentiation and electrical activity measurement over 7 WIV (left). The differentiation of oligodendrocytes requires a long-term differentiation protocol. HiNPC neurospheres were differentiated in a shaking incubator for 8 WIV to obtain BrainSpheres, which consist of neurons, astrocytes and oligodendrocytes (right). **C** Immunoblotting of CSB levels in neurospheres of the four hiNPC lines, normalized to the loading control HSP90 and to the respective control (left) or IMR90^WT^ (right). Representative images are depicted and graphs visualize differences in protein content due to mutations in the CSB gene or between CSB expressing lines, respectively. Graphs depict N = 3 biological replicates, mean ± SEM. Statistical analyses were performed using unpaired two-tailed t-tests. A p-value below 0.05 was termed significant. **D** HiNPC spheres were plated, differentiated for 3DIV and subsequently stained for nuclei (Hoechst, blue), neurons (MAP2, yellow) and astrocytes (AQP4, magenta). **E** Transcriptome analyses were performed by plating hiNPC neurospheres onto a coated surface, and differentiating these for 3, 14 and 21 DIV. VENN Diagram of the significantly DEGs between IMR90^WT^ and IMR90^KO^ after 3, 14 and 21 DIV, as identified via RNAseq. DEG criteria: |log2| > = 1 and Qvalue of <  = 0.05. **F** 893 DEGs constantly regulated across the differentiation timepoints were mapped to KEGG pathways and the 30 pathways with the highest percentage of regulated genes are depicted. Abbreviations: *hiPSC* human induced pluripotent stem cells, *hiNPCs* human induced neural progenitor cells, *MEA* microelectrode array (MEA), *WIV* weeks in vitro, *DIV* days in vitro, *DEG* differentially expressed gene, *RNAseq* RNA Sequencing

For investigating the CSB neuropathology, we established two distinct hiPSC disease models, each including a CSB-deficient and a CSB-expressing cell line. The first model consists of the commercially available hiPSC line IMR90^WT^, which expresses the CSB wildtype in a healthy donor genetic background and an IMR90^KO^ line generated via CRISPR Cas technology, by introducing a 13 bp deletion into exon 5 of the ERCC6 gene (Fig. 1A, left; supplemental information (SI) Fig. S1). The second model consists of the patient-derived hiPSC line CS789 and the isogenic control line CS789^Res^. The patient cell line holds a point mutation (2047C > T) in exon 10 of the ERCC6 gene, which leads to a premature stop codon. The genetic rescue of the control line (CS789^Res^) was generated via CRISPR Cas technology, through a 9 bp in-frame deletion, which removes the stop codon (Fig. 1A, right; SI Fig. S1). The donor of the patient hiPSCs was classified with Cerebro-oculofacio-skeletal syndrome (COFS), one of the most severe forms of CSB (supplemental information (SI) Table S1). The patient was, amongst others, afflicted with congenital microcephaly, severe intellectual disability and seizures. He passed away at 10 months of age.

The hiPSC lines were neurally induced into neurospheres containing hiNPCs, and subsequently either differentiated on an artificial extracellular matrix or floating as 3D BrainSpheres into neurons, astrocytes and facultatively oligodendrocytes (Fig. 1B), thereby generating different fit-for-purpose models.

First, we confirmed by western blot, that the CSB protein is expressed in hiNPC neurospheres of both healthy cell lines (IMR90^WT^ and CS789^Res^), but not in either of the disease cell lines IMR90^KO^ and CS789 (Fig. 1C left). Despite varying levels of expression (Fig. 1C right), the CSB protein was detected in both IMR90 and CS789^Res^ lysates. Due to the non-isogenic nature of the two lines, such differences in expression levels of the CSB protein can be expected. We next confirmed that hiNPC neurospheres of all four cell lines are able to differentiate into cells of the neural lineage, containing both neurons and astrocytes (Fig. 1D).

To obtain an understanding of the consequences of CSB-deficiency for the hiNPCs’ bulk transcriptome, we performed mRNA Sequencing (RNAseq) analyses. Here we decided for the control IMR90^WT^ and the IMR90^KO^ lines, in order to understand the effects of the clean CSB mutation and to exclude impacts of the patient specific genetic background. Transcriptome analyses were performed by plating hiNPC neurospheres onto a coated surface, and differentiating these for 3, 14 and 21 days in vitro (DIV). In total, 893 genes were differentially expressed in IMR90^KO^ compared to IMR90^WT^ cells across all time points (Fig. 1E). Only significantly regulated genes with a |log2| of ≥ 1 were analyzed. These differentially expressed genes (DEG) were mapped to Kegg pathways and the top regulated pathways are depicted in Fig. 1F. Most DEGs map to autophagy (ID4140) and mitophagy (ID4137), as well as associated pathways such as MAPK (ID4010), RAP1 (ID4015), PI3K (ID4151) and Ras (ID4014) signaling. Another high number of DEGs map to Cell adhesion molecules (CAMs, ID4514), the regulation of the actin cytoskeleton (ID4810) and focal adhesions (ID4510), as well as calcium signaling (ID4020) and glutamatergic synapses (ID4724).

### CSB-deficiency is associated with inhibited neural progenitor cell migration and alterations in focal adhesion and autophagy

**Fig. 2:**
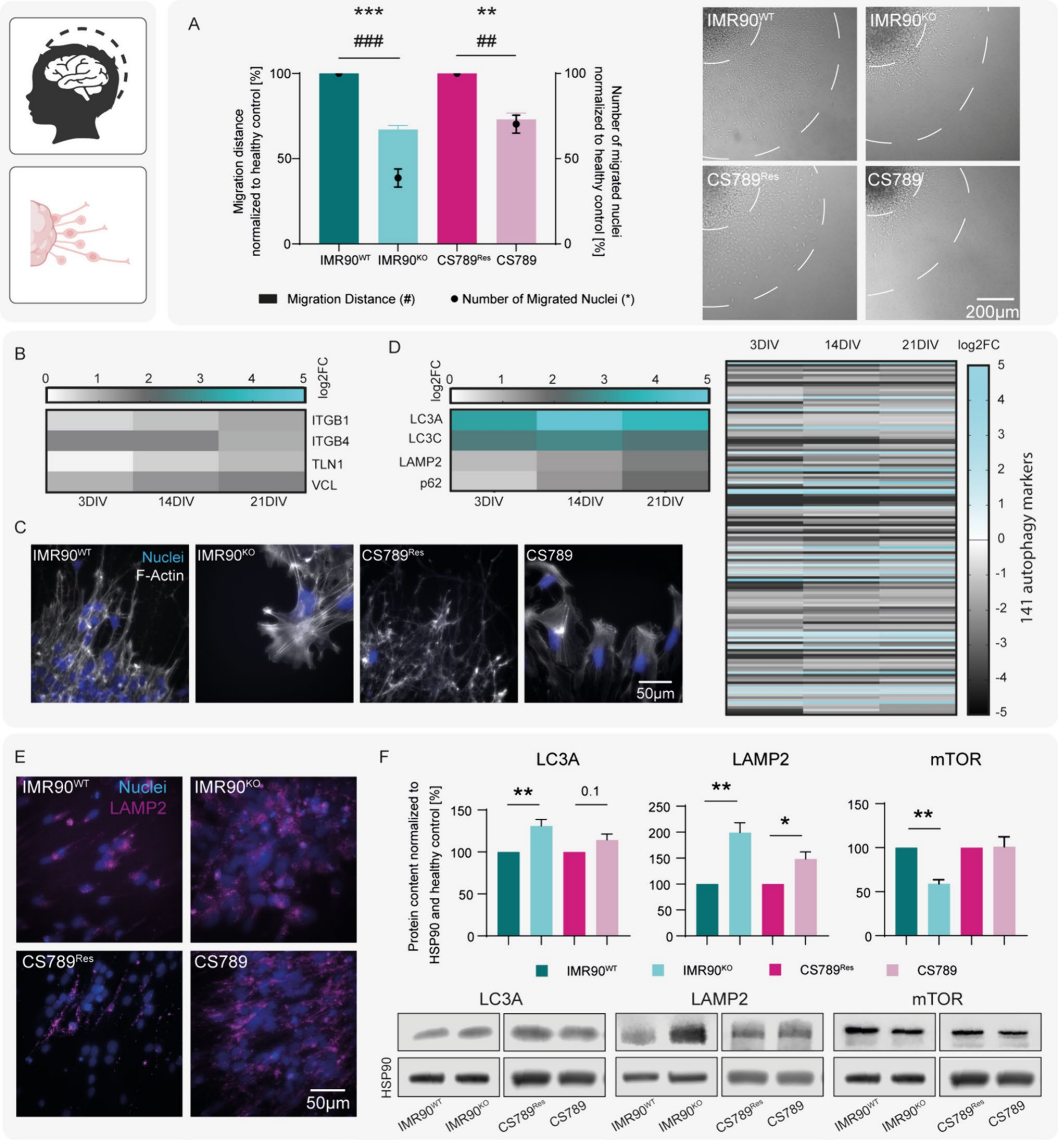
CSB-deficiency is associated with inhibited neural progenitor cell migration and alterations in focal adhesion and autophagy. **A** 0.3 mm hiNPC neurospheres were plated and differentiated for 3 DIV. Cell migration was assessed by measuring both migration distance of furthest migrated cells (bars) and the total number of migrated nuclei (dots). Exemplary brightfield images are shown on the right. The graph depicts N = 3 biological replicates with n = 5–10 spheres each, mean ± SEM. **B** The heat map shows selected significantly regulated DEGs of focal adhesion-related pathways, identified using RNA Sequencing in the IMR90^WT^ and IMR90^KO^ cell lines. DEG criteria: |log2| > = 1 and Qvalue of <  = 0.05. **C** Representative stainings of the migration area of plated hiNPC neurospheres after 3 DIV, showing nuclei (blue) and the cytoskeletal marker F-actin (white). **D** Heatmap of the 140 differentially regulated autophagy-associated genes of IMR90^KO^ compared to IMR90^WT^ acoss 3, 14 and 21 DIV as identified using RNA Sequencing (right), and separately highlighted DEGs of interest (left). DEG criteria: |log2| > = 1 and Qvalue of <  = 0.05. **E** Representative ICC images of differentiating hiNPC neurospheres after 3DIV, showing nuclei (blue) and lysosomes (LAMP2, magenta). **F** Western Blot of plated and 3DIV differentiated hiNPC spheres, quantifying the fold change in LC3A, LAMP2 and mTOR protein levels, normalized to the loading control HSP90 and the respective CSB expressing control cell line. Graphs depict N = 3 biological replicates, mean ± SEM. For **A** and **F** a p-value below 0.05 was termed significant. Statistical analyses were performed using unpaired two-tailed t-tests. Abbreviations: *NPCs* neural progenitor cells, *DEG* differentially expressed gene, *DIV* days in vitro, *ICC* Immunocytochemistry

Microcephaly is a major neuropathological finding in CSB patients [1, 7]. Two underlying cellular deficiencies that might cause microcephaly are inhibited NPC proliferation or migration [49, 50]. Therefore, we investigated these neurodevelopmental KEs in the CSB proficient and deficient in vitro models. Proliferation was assessed in hiNPC neurospheres, by measuring the sphere diameter over time, and by quantifying the incorporation of Bromodeoxyuridine (BrdU) into newly synthetized DNA. No significant differences between healthy and disease neurospheres were observed in either proliferation assay (SI Fig. S2). Next, we assessed the migration capacity of plated hiNPC neurospheres by measuring total migration distance from the rim of the sphere core to the furthest cell that migrated out of the sphere core, and by counting the total number of migrated nuclei after 3 DIV within the migration area. We observed a significant reduction of the migration distance (25–35%), and a significantly decreased number of migrated nuclei (25–60%) within the migration area using hiNPCs from disease cell lines, compared to their respective controls (Fig. 2A). In support of the inhibited migration, transciptome analyses present significant upregulation of the focal adhesion-specfic gene expression markers *Integrin ß1* (*ITGB1*), *Integrin ß4* (*ITGB4*), *Talin-1* (*TLN1*) and *Vinculin* (*VCL*) in the IMR90^KO^, compard to the healthy IMR90^WT^ hiNPC neurospheres after 3, 14 and 21 DIV (Fig. 2B). An important intracellulart part of focal adhesions during migration are actin stress fibers. In line with the altered expression of genes involved in regulating the actin cytoskeleton (Fig. 1G), we observed thickened and elongated actin stress fibers in the migration area of plated disease hiNPC neurospheres after 3DIV (Fig. 2C). Inhibited focal adhesion turnover and subsequent migration inhibition thus seems to be one in vitro feature of CSB-deficiency.

In addition to the actin- and focal adhesion-related genes, 141 autophagy-associated mRNAs are significantly regulated in the disease IMR90^KO^, compared to IMR90^WT^ differentiated neurospheres (Fig. 2D, right). Gene expression of the autophagosome markers *microtubule-associated proteins 1A/1B light chain 3A* (*LC3A*) and *3C* (*LC3C*) are highly upregulated in the disease differentiated neurospheres. Additionally, the lysosome marker *lysosomal-associated membrane protein-2* (*LAMP2*) and *p62*, a gene coding for a protein that taggs intracellular material for targeted autophagy, are also upregulated in the disease differentiated neurospheres (Fig. 2D, left). On the protein level, we identified LAMP2 accumulation in immunocytochemical stainings (ICC, Fig. 2E) and confirmed significant LC3A and LAMP2 accumulation in both CSB-deficient IMR90^KO^ and CS789 lines by Western Blot (WB) analyses of plated hiNPC neurospheres after 3 DIV (Fig. 2F). The accumulation of these proteins is a general marker for defective autophagy. Futhermore, a decreased level of mammalian target of rapamycin (mTOR), an autophagy-inhibitor, in IMR90^KO^ hints towards an increased demand for autophagy in the disease cells (Fig. 2F).

### The HDAC6-inhibitor Tubastatin A partially rescues the migration phenotype of disease hiNPC neurospheres

**Fig. 3:**
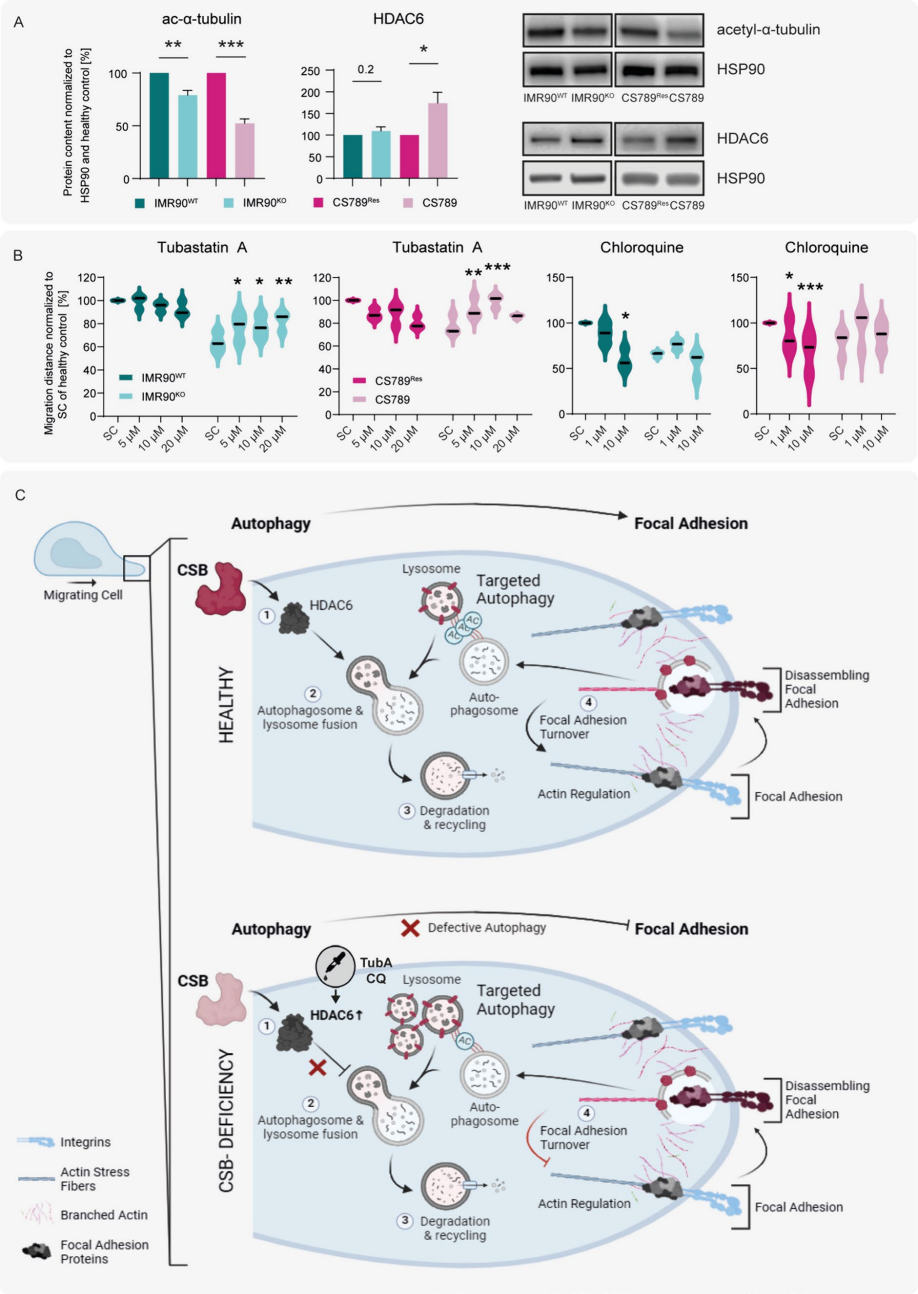
HDAC6-inhibitor Tubastatin A partially rescues the adverse migration phenotype of CSB-deficient cells. **A** Exemplary images and quantifications of Western Blot analyses depicting the fold change in acetyl-alpha-tubulin and HDAC6 protein levels, normalized to the loading control HSP90 and the respective control cell line. Graphs depict N = 3 biological replicates, mean ± SEM. **B** 0.3 mm hiNPC neurospheres were plated and differentiated under exposure to TubA (left) or CQ (right), before the migration distance was measured on DIV 3. The migration distance of both healthy and disease cell lines was normalized to the solvent control (SC) of the respective healthy cell line within each graph. Viability and cytotoxicity were assessed in parallel (Supplemental Information Figure S3). Graphs depict N = 3 biological replicates with n = 5–10 spheres each. **C** Illustration of the presumed CSB mechanism: CSB-deficiency correlates with increased HDAC6 expression (1) and reduced a-tubulin acetylation. This causes defective autophagy, specifically impaired autophagosome and lysosome fusion (2) and degradation (3) and hence lysosome accumulation. This in turn inhibits migration through inefficient focal adhesion turnover and altered regulation of the actin cytoskeleton (4). This cellular disease phenotype can be partially rescued by Tubastatin A and Chloroquine exposure of CSB-deficient cells. For **A** and **B**, a p-value below 0.05 was termed significant. Statistical analyses in **A** were performed using unpaired two-tailed t-tests and in **B** using one-way ANOVA with post-hoc Dunnett tests. Abbreviations: *NPCs* neural progenitor cells, *TubA* Tubastatin A, *CQ* Chloroquine. Figure created with biorender.com

A previous study on CSB-deficient fibroblasts suggested CSB to interact with α-tubulin acetyltransferase MEC-17 and histone deacetylase 6 (HDAC6). MEC-17 acetylates α-tubulin, thereby supporting cargo transport and autophagosome with lysosome fusion, while HDAC6 mediates histone deacetylation [18]. We observed significantly decreased acetyl-α-tubulin levels and increased HDAC6 levels in differentiated and plated disease hiNPC neurospheres after 3DIV, compared to their respective healthy controls also after 3DIV (Fig. 3A). Low levels of α-tubulin acetylation generally suggest inhibited autophagy in the cell.

Since targeted autophagy is an important facilitator of focal adhesion turnover, which in turn enables cell migration, we performed HDAC-inhibition experiments to link HDAC activity to altered migration of the CSB disease models. Therefore, we treated the migrating hiNPC spheres at the time of plating with the pan-HDAC inhibitor suberoylanilide hydroxamic acid (SAHA) or the HDAC-6-specific inhibitor Tubastatin A (TubA) for 3 DIV. SAHA did not significantly rescue the migration of the CSB-deficient cell lines, yet reduced the cell viability significantly even at low concentrations (SI Fig. S3). Treatment with TubA, however, successfully rescued the reduced migration in both IMR90^KO^ and CS789 disease lines, at non-cytotoxic concentrations (Fig. 3B left, see also SI Fig. S3). To further strengthen the causal link between autophagy and migration, we inhibited the autophagy in migrating cells with chloroquine (CQ), a suppressor of autophagosome and lysosome fusion. Our results show a significant migration inhibition at non-cytotoxic CQ concentrations in both healthy cell lines (Fig. 3B right, SI Fig. S3). Since autophagy is already compromised in the disease cell lines, CQ does not further reduce migration of IMR90^KO^ or CS789 at non-cytotoxic concentrations. Considering these results, we propose a putative CSB mechanism: CSB-deficiency correlates with increased HDAC6 expression and reduced a-tubulin acetylation. This causes defective autophagy, specifically impaired autophagosome-lysosome fusion and degradation and thus lysosome accumulation. This in turn inhibits migration through inefficient focal adhesion turnover and altered regulation of the actin cytoskeleton (Fig. 3C).

### Microelectrode array measurements reveal altered neural network activity in CSB-deficient networks

**Fig. 4:**
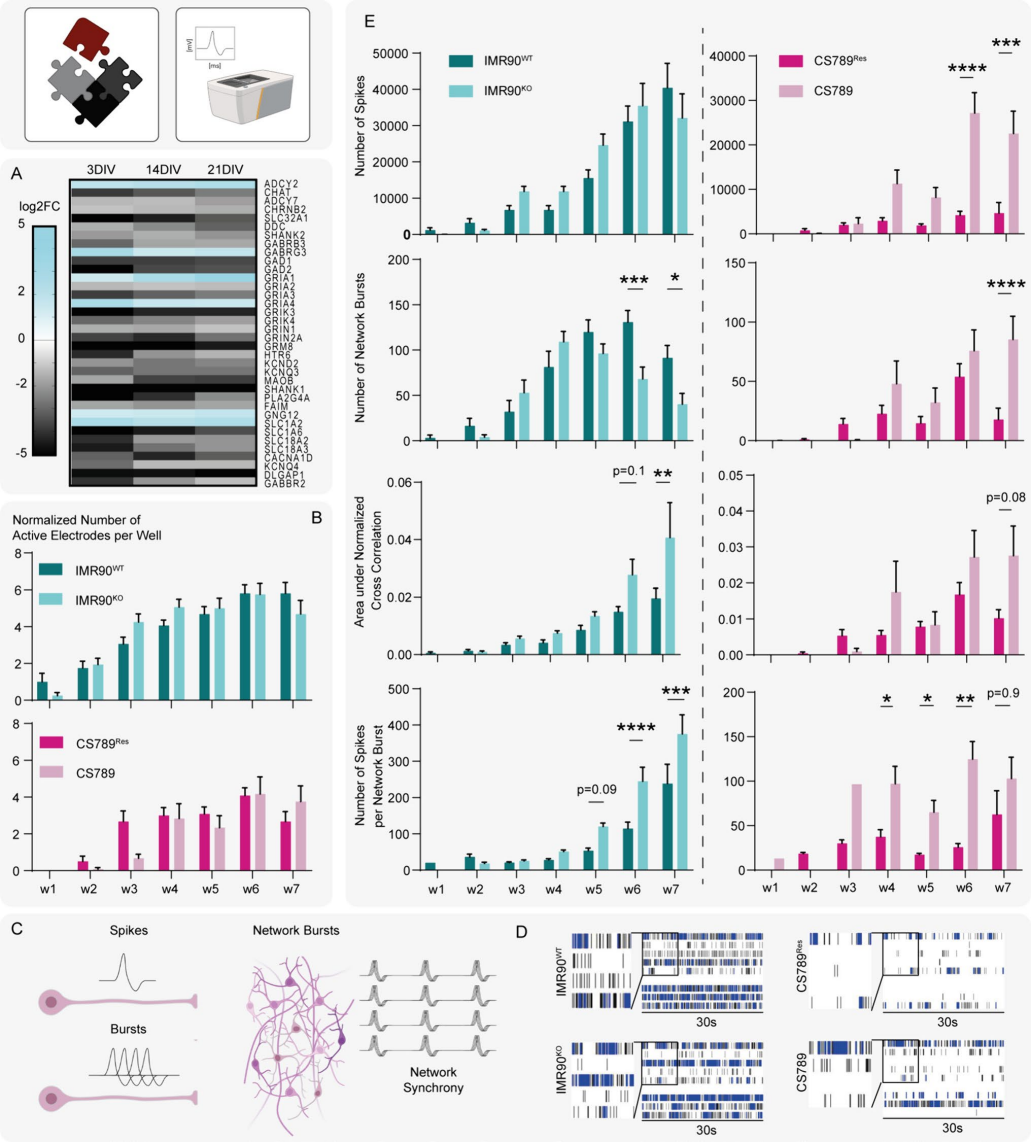
Microelectrode Array measurements reveal altered neural network activity in CSB-deficient networks. **A** Heatmap of the 36 differentially regulated synapse-associated genes of IMR90^KO^ compared to IMR90^WT^ accoss 3, 14 and 21 DIV. DEG criteria: |log2|> = 1 and Qvalue of <  = 0.05. **B** HiNPC neurospheres were plated onto MEA plates (96-well, 8 electrodes per well) and differentiated for neural network formation. The neural network development was assessed by measuring the electrical signals over 7 weeks. The graphs depict the number of active electrodes normalized to the number of wells over time (N = 8–12 wells with n = 64–96 electrodes each; mean ± SEM). **C** Illustration of the MEA-derived parameters single spikes, bursts, network bursts and network synchronicity. **D** Representative spike raster plots (SRP) of one well of each cell line after 6 WIV. Displayed are the spikes (black) and bursts (blue) of the first 30 s of the 15 min total measurement. **E** The electrical activity of neural networks over time is depicted and compared to the respective control. The graphs show the mean ± SEM of three biological replicates for the selected parameters number of spikes, number of network bursts, area under normalized cross correlation (network synchronicity) and number of spikes per network burst (per replicate 8–12 wells with 64–96 electrodes were evaluated). Each time point comprises a 15 min measurement. A p-value below 0.05 was termed significant. Statistical analyses of MEA results were performed using Mixed-effects analysis with the Sidak test for multiple comparisons. Abbreviations: *NPCs* neural progenitor cells, *MEA* microelectrode array. Figure created with biorender.com

Next, we investigated the ability of the CSB-deficient cell lines to form functional neural networks as a possible pathomechanism for the intellectual disability seen in CSB patients [1, 2, 7, 51]. These studies were inspired by the transcriptome analyses, which revealed strong alterations in the expression of genes involved in synapse formation, neurotransmitter synthesis and signaling (Fig. 4A). A number of genes involved in the biosynthesis (*CHAT, DDC, GAD1, GAD2*) and vesicular transport (*SLC32A1, SLC18A2, SLC18A3*) of neurotransmitters are significantly downregulated in IMR90^KO^, compared to IMR90^WT^. Genes for postsynaptic glutamate receptors (*GRIA2, GRIA3, GRIA4, GRIK4, GRIN1, GRIN2A, GRM8*), GABA receptors (*GABRB3*), acetylcholine receptors (*CHRNB2*) and serotonin receptors (*HTR6*) are significantly downregulated in the CSB-deficient cells, while few genes for glutamate and GABA receptors are significantly upregulated (*GRIA1, GRIK3, GABRG3*). Further differentially expressed genes are involved in postsynaptic density (*SHANK1, SHANK2, DLGAP1*), voltage-gated (*KCND2, KCNQ3, PLA2G4A, CACNA1D, KCNQ4*) and G-protein coupled (*ADCY2, ADCY7, GNG12*) signaling and neurotransmitter cycling (*MAOB, SLC1A2, SLC1A6*). Gene markers *MAP2* for neurons, *SYN* for pre-synapses and *PSD95* for post-synapses are not significantly differentially expressed (SI Fig. S4).

To get a functional readout of the neural network formation, we recorded the electrical activity of neural networks derived from all cell models on microelectrode arrays (MEA). HiNPC neurospheres were plated onto 96-well MEAs and differentiated for neural network formation over a period of 7 weeks. This allows us to follow the formation of neural networks over time. Using MEAs, we can analyze multiple parameters, that are spike- burst- and network-related and represent single neuronal activity, single neuronal and network maturation, respectively (Fig. 4C) [52]. The number of active electrodes increased in all neural networks and plateaued at approx. 4–5 active electrodes per well (Fig. 4B). Representative spike raster plots (SRP) are shown in Fig. 4D. The SPR of each cell line depicts the spikes (black) and bursts (blue) of the first 30 s of the 15 min measurement after 6 weeks in vitro (WIV). We observed differences in some of the measured MEA parameters, between CSB-deficient and -proficient networks, i.e. number of spikes, number of network bursts, area under normalized cross correlation (network synchrony) and number of spikes per network burst (Fig. 4E). The IMR90^KO^ networks show no significant difference in the number of spikes compared to the IMR90^WT^ networks. However, a significant reduction of network bursts and a significant increase in area under normalized cross correlation and number of spikes per network bursts can be seen, starting from 5 WIV. Similarly, CS789 shows significantly increased area under normalized cross correlation and number of spikes per network bursts. However, the parameters ‘number of network bursts’ and ‘number of spikes’ are both significantly increased in CS789 compared to its control CS789^Res^. Although the two different models cannot be directly compared, alterations on neural networks are similar, both indicating an increased electrical activity of the disease cell lines compared to their healthy controls. We observed interesting differences regarding the number of network bursts between the two genetic backgrounds in the IMR90WT/IMR90KO and CS789/CS789Res systems that need to be investigated in follow up studies. The significant changes in electrical activity of both model systems hint towards an altered neural network formation in the disease system and hence are in line with the transcriptome results. Noteworthy are especially the alterations of IMR90^KO^ and CS789 in the network activity, compared to their respective controls. Rescue trials with above mentioned HDAC-inhibitors TubA and SAHA did not change the neural network activities of either network (SI Fig. S5).

### Altered GABA levels and KCC2 expression hint towards a delayed GABA switch in disease cell lines

**Fig. 5:**
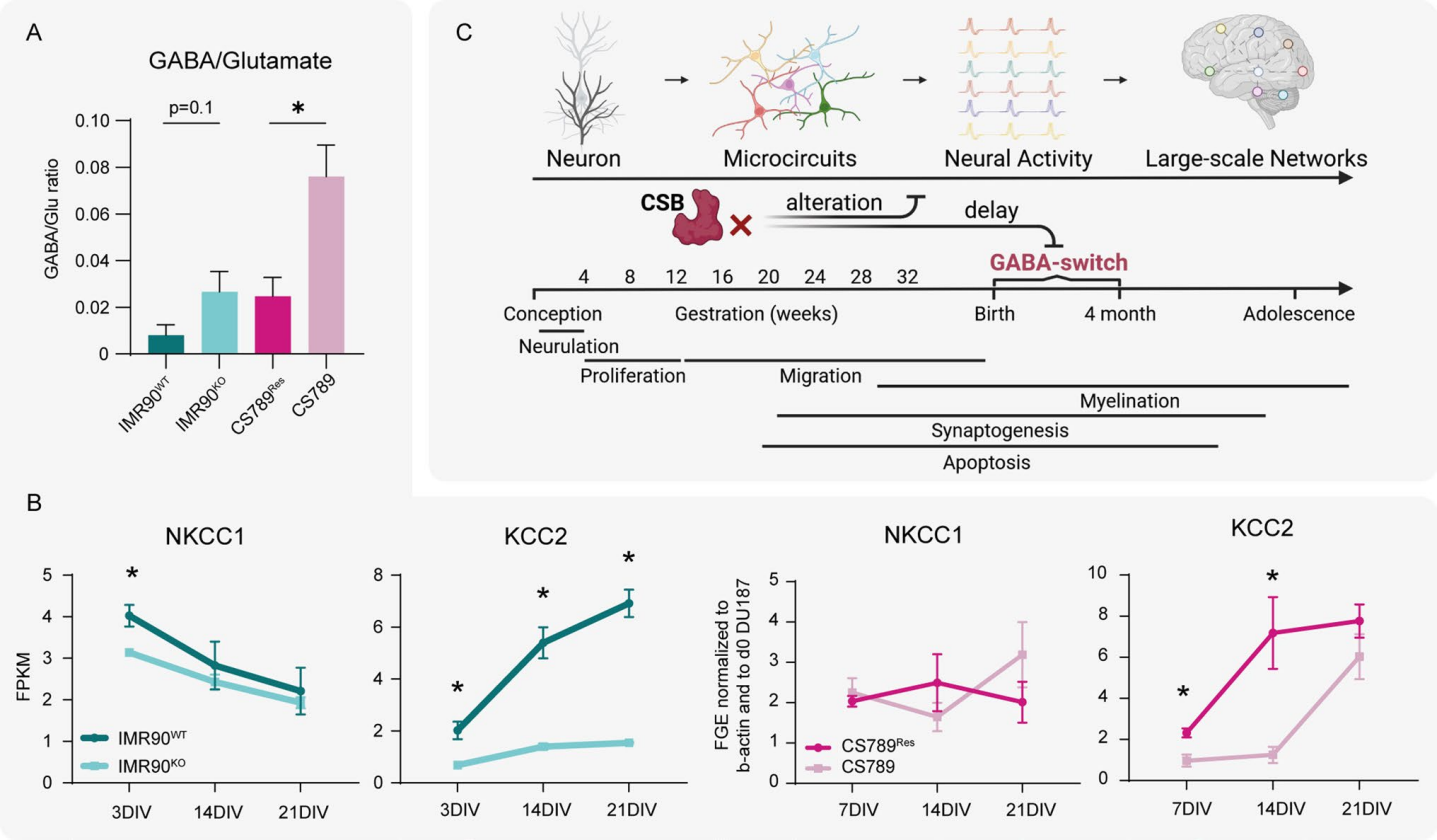
Elevated GABA/Glutamate ratios and delayed KCC2 induction hint towards a delayed GABA switch in CSB-deficient cells. **A** HiNPC neurospheres were plated and differentiated for 14 DIV, before GC–MS was performed. Graphs depict the ratios of the relative metabolite concentrations of neurotransmitters glutamate and GABA (N = 3 biological replicates, mean ± SEM). **B** Left: Differential expression of *NKCC1* and *KCC2* in IMR90^WT^ and IMR90^KO^ was evaluated from RNA sequencing data. Graphs depict the FPKM raw values over 21 DIV. Right: *NKCC1* and *KCC2* mRNA expression in CS789^Res^ and CS789 was analyzed via qPCR. Graphs depict the FGE over 21 DIV, normalized to β-actin and to d0 of CS789^Res^ (not shown; N = 3 biological replicates, mean ± SEM). **C** Illustration of the presumed effects of CSB-deficiency on the brain circuit formation, possibly through GABA-switch delay. A p value below 0.05 was termed significant. Statistical analyses were performed using unpaired two-tailed t-tests. Abbreviations: *DIV* days in vitro, *FPKM* fragments per kilobase million, *FGE* fold gene expression GC–MS, gas chromatography–mass spectrometry. Figure adapted from Tau et al. [87] created with biorender.com

In order to evaluate, whether the increase of spiking in CSB-deficient neural models is caused by excitatory neurotransmitter accumulation, we performed Mass Spectrometry (MS) analyses. For that hiNPC neurospheres were plated and differentiated for 14 DIV. Excitatory glutamate levels and GABA levels, show no significant difference between disease and control neurospheres (SI Fig. S6 and the supplementary SI Table S4). However, their respective GABA/glutamate ratios, calculated from the relative metabolite concentrations, show an accumulation of GABA in both CSB-deficient systems, with significant accumulation in CS789 compared to CS789^Res^ (Fig. 5A). The neurotransmitter GABA has excitatory consequences before the postnatal GABA-switch, and holds inhibitory functions thereafter. The transition of this GABA switch is initiated by crucial changes in gene expression of the chloride importer *Na–K–2Cl cotransporter isoform 1* (*NKCC1*) and the exporter *K–Cl cotransporter isoform 2* (*KCC2*). During the GABA-switch, *NKCC1* expression decreases, while *KCC2* expression increases. As active chloride transporters, they are responsible for the high and low concentrations of intracellular chlorine before and after GABA switch, respectively [53]. The control IMR90^WT^-derived neural networks mimic this development over 21 DIV with increasing *NKCC1* and decreasing *KCC2* expression. In contrast, *KCC2* is not upregulated in the CSB-deficient networks derived from IMR90^KO^, indicating a disturbed GABA switch (analyzed via RNA Sequencing, Fig. 5B left). A similar picture is observed in the CS789^Res^ and CS789 model, yet the GABA switch seems rather delayed, as *KCC2* expression is only significantly lower during the first 14 DIV (analyzed via qPCR, Fig. 5B right). *NKCC1* expression does not change over 21 DIV for this system.

### CSB-deficiency leads to hindered oligodendrocyte maturation

**Fig. 6:**
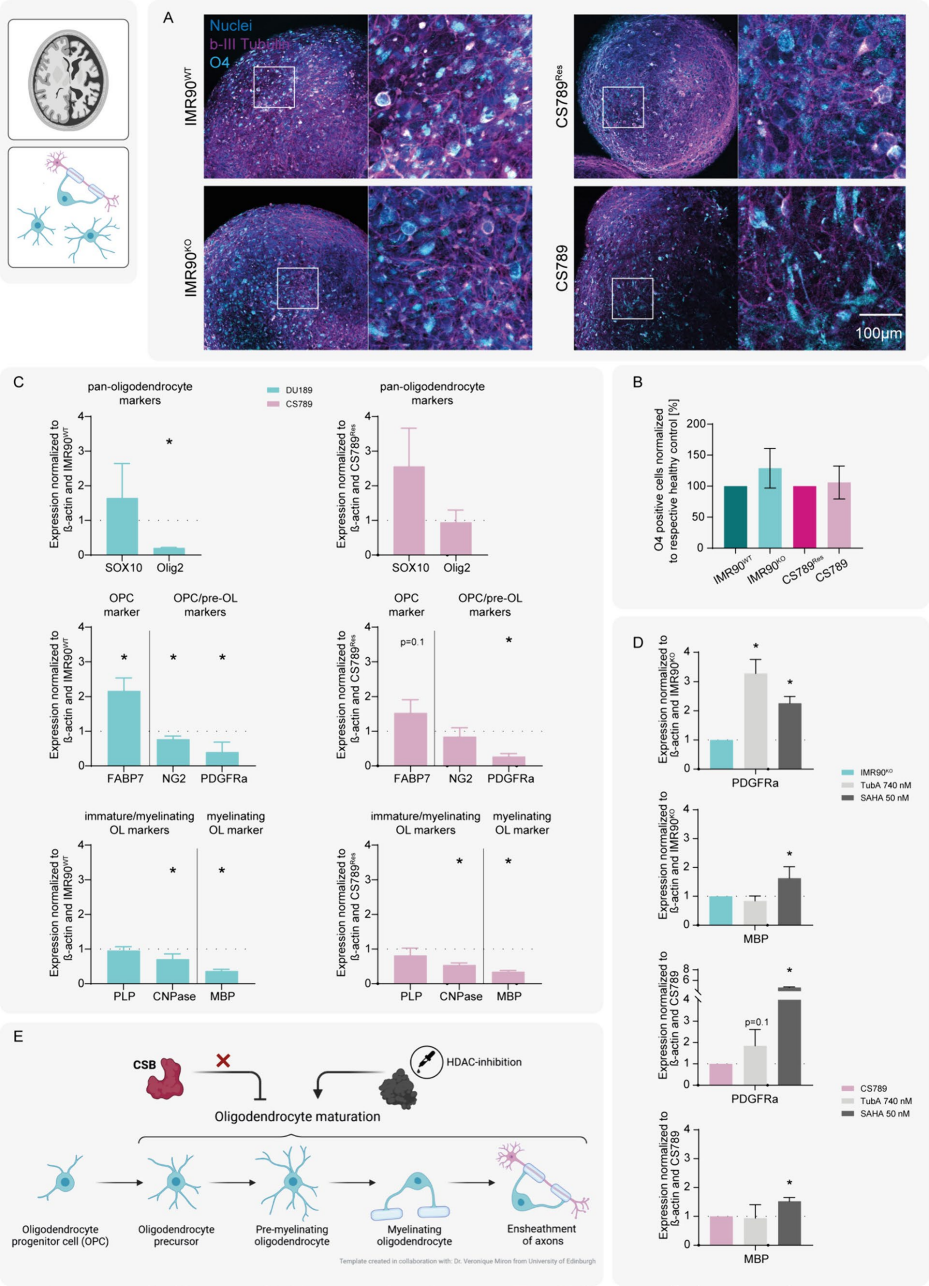
CSB-deficiency leads to hindered oligodendrocyte maturation. HiNPC neurospheres were pre-differentiated in designated oligodendrocyte differentiation medium for 8 weeks in an orbital shaking incubator to generate oligodendrocyte-containing BrainSpheres. **A** Representative 3D BrainSphere ICC stainings after 8 WIV, showing nuclei (Hoechst, blue), neurons (β-III tubulin, magenta) and oligodendrocytes (O4, cyan). **B** Quantification of O4 positive cells after 4 weeks of adherent oligodendrocyte differentiation, normalized to the respective healthy control cell line. **C** Analyses of oligodendrocyte marker mRNA expression via qPCR after 8 WIV. Left: Expression in IMR90^KO^ compared to IMR90^WT^. Right: Expression in CS789 compared to CS789^Res^. Graphs depict N = 3 biological replicates, mean ± SEM. **D** After 6 WIV IMR90^KO^ and CS789 BrainSpheres were treated with 250 nM TubA or 50 nM SAHA for two weeks, while remaining in the orbital shaking incubator. The graphs show marker gene expression at 8 WIV as assessed by qPCR. All graphs depict N = 3 and mean ± SEM. Marker gene expression is normalized to β-actin and respective expression in IMR90^WT^. **E** Illustration of the presumed effect of CSB-deficiency on HDAC-dependent oligodendrocyte maturation. A p-value below 0.05 was termed significant. Statistical analyses were performed using unpaired two-tailed t-tests. Abbreviations: *NPCs* neural progenitor cells, *WIV* weeks in vitro, *ICC* Immunocytochemistry, *TubA* Tubastatin A. Figure created with biorender.com

Demyelination is a common neuropathological phenotype of CSB patients [1, 2, 7, 14, 51]. Generating hiPSC-based BrainSpheres containing oligodendrocytes in addition to neurons and astrocytes in 3D requires a long-term protocol, that cultures the floating spheres in a shaking incubator (Fig. 1B). Here, BrainSpheres provide a more mature model compared to hiNPC neurospheres, due to their longer maturation period in 3D. After 8 weeks, BrainSpheres were analyzed by immunocytochemical staining for neurons (β-III-tubulin) and oligodendrocytes (O4; Fig. 6A). No obvious morphological or numerical differences were observed in O4-stained oligodendrocytes differentiated from disease and respective control hiPSCs. In addition, hiNPC neurospheres were plated for subsequent adherent oligodendrocyte differentiation. After 4 weeks, cells were stained for the pan-oligodendrocyte marker O4 and scanned with a high-content imaging platform. Scanned images were then analyzed for the number of O4 positive cells, using a colocalization tool, which colocalizes nuclei and O4 staining in ICC images (Fig. 6B and SI Fig. S7). No significant differences in O4 staining was detected. Myelin formation is dependent on oligodendrocyte differentiation and their maturation. Therefore, we explored if CSB-deficiency impacts oligodendrocyte maturation, by analyzing the gene expressions of pan- and maturation stage-specific oligodendrocyte markers (Fig. 6C). No significant difference in the expression of the pan-oligodendrocyte marker *SOX10* between CSB-proficient and -deficient BrainSpheres was observed. A second pan-oligodendrocyte marker, *Olig2*, was expressed significantly less in BrainSpheres from IMR90^KO^, compared to IMR90^WT^, yet this was not seen in CS789 compared to CS789^Res^. The oligodendrocyte progenitor cell (OPC) marker *FABP7* was overexpressed in both, IMR90^KO^ (significantly) and CS789-derived BrainSpheres. The OPC and pre-oligodendrocyte (pre-OL) markers *NG2* and *PDGFRa* were both underexpressed in IMR90^KO^, while CS789 underexpressed *PDGFRa*, but not *NG2*. Correspondingly, expression of the immature and myelinating OL markers *CNPase* and *MBP* were underrepresented in both CSB-deficient BrainSpheres, compared to their respective controls. *PLP* was not differentially expressed in either cell system. These results point to a delayed maturation of developing oligodendrocytes in the CSB-deficient models, with an overexpression of the OPC-specific gene FABP7 and an underexpression of genes specific for more mature oligodendrocyte stages.

In the next step we assessed, whether this impaired oligodendrocyte maturation is mediated by HDACs, similar to the CSB consequences on migration. Between 6 to 8 WIV, a time where oligodendrocyte maturation takes place in BrainSpheres, IMR90^KO^ and CS789 BrainSpheres were treated with 740 nM TubA or 50 nM SAHA (Fig. 6D). Both HDAC inhibitors significantly antagonized the CSB deficiency-dependent underexpression of the pre-OL marker *PDGFRa* in IMR90^KO^ and CS789. SAHA additionally induces a significant increase in the expression of the myelinating OL marker *MBP* in both CSB-deficient BrainSpheres (Fig. 6E).

## Discussion

Mimicking human disease and identifying treatments with animal models often undermines expectations. Especially for diseases involving the brain, translation from animals to humans is challenging. Species differences in brain physiology and kinetic properties are key here, with high dropout rates in drug development pointing to this [54]. Drugs developed for CNS diseases display the second highest attrition rates right after cancer drugs with causes of drug failure allocating to lack of efficacy and second most frequently to toxicity [55]. As an example, drug development for treating Alzheimer’s disease alone produced over 99% failure rates [56]. Similarly, treatments for neurodevelopmental disorders like autism spectrum disorders [57] are sparse. This is mainly due to the lack of pathophysiological understanding of the disease and a consecutive lack of known drug targets. In this study we aim at setting an example for unraveling molecular and cellular causes of a severe neurodevelopmental disease, the Cockayne Syndrome B (CSB), using 3D neural models like hiPSC-derived neurospheres and BrainSpheres. We identified in vitro phenotypes that we relate to the children’s pathophysiology and based on that propose novel treatment strategies for this devastating disease.

CSB is a heterogeneous hereditary disease with a spectrum of clinical phenotypes highly depending on the associated mutant genotype. However, common pathophysiological brain features of CSB patients include microcephaly, intellectual disability and demyelination [1, 2, 7, 14]. In this work, we provide for the first time mechanistic explanations for the cardinal brain phenotypes observed in CSB patients. We here use two 3D hiPSC-derived neural CSB models and their isogenic controls, a CSB patient-derived line and a genome-edited healthy donor hiPSC line carrying a truncating CSB mutation, both of which result in CSB protein deficiency. Specifically, our results suggest that CSB deficiency inhibits migration through defective autophagy, which is consistent with the clinical microcephaly observed in CSB patients. Further observations of altered electrophysiology and changes in GABA neurotransmitter levels in CSB-deficient neural networks indicate that a disturbed GABA switch is involved in altered brain circuit formation, ultimately leading to intellectual disability in patients. In addition, the impaired oligodendrocyte maturation we observed in CSB-deficient BrainSpheres provides an explanation for the demyelination observed in children with CSB. Therefore, using human-based 3D in vitro models, we identified multiple cellular pathomechanisms of CSB deficiency and were able to link them to the three cardinal brain phenotypes of CSB patients.

Microcephaly can be caused by dysfunctional NPC proliferation, migration, neuronal differentiation or apoptosis [49, 50]. We are the first to show that deficiency of the CSB protein causes NPC migration defects. Others previously reported on disrupted neuronal differentiation and neurite outgrowth as a consequence of CSB-deficiency in 2D immortalized NPC and hiPSC-derived neurons, respectively [30, 31], which might also contribute to the microcephalic CSB phenotype. We did not observe an impairment of neuronal differentiation in the differentiating CSB-deficient neurospheres (SI Fig. S4), which could be explained by the large heterogeneity of CSB phenotypes between patients. Yet, these differences might also arise due to the different cell systems (immortalized NPCs vs. hiNPCs) or dimensionality (2D vs. 3D). Next, we substantiated the findings of impaired migration by mechanistic understanding. The disrupted migration of CSB-deficient hiNPC neurospheres is accompanied by altered markers of autophagy, i.e. dysregulated auto- and mitophagy-related gene and protein expressions and a reduced amount of acetylated α-tubulin. Disrupted autophagy based on malfunctioning HDACs was recently established as a major mechanism in the skin pathology of CSB patients [18]. Here we extend this mechanism from the skin to the developing brain, by showing that the HDAC6-specific inhibitor Tubastatin A rescues the inhibited migration in the CSB-deficient neurospheres. This leads us to hypothesize that HDAC-dependent defective autophagy is the cause for the impaired migration. This hypothesis is supported by the well-established knowledge that targeted autophagy plays a major role in focal adhesion turnover, which in turn facilitates cell migration (Fig. 3C) [58–60]. HDAC-inhibitors can have highly context-dependent off-target effects, such as decreased proliferation, increased cell death or histone modifications, which need to be evaluated individually, ideally in an organism-specific manner. However, selective HDAC-inhibitors are expected to have a high molecular specificity [61].

Besides migration, we also studied the functionalities of developing CSB-proficient and -deficient neural networks over 7 WIV on MEAs, as they provide a promising tool to investigate disease-associated alterations in neural circuit formation in vitro [62, 63]. Brain circuit formation is precisely orchestrated during brain development and a disruption leads to numerous pathological defects, many of which culminate in intellectual disabilities [64–66]. Mutations in the CSB spectrum are also frequently accompanied by limited cognitive function and delayed neurodevelopment [1, 2, 7], leading us to speculate that disrupted circuit formation might be one of the underlying causes. Both CSB-deficient in vitro neural networks show increased and/or accelerated electrophysiological parameters compared to their healthy controls over time. The differences seen in some parameters when cross-comparing the two models may arise from the individual properties of the distinct cell lines, which underlines the necessity of isogenic controls for such disease models. In addition, neurotransmitter analyses coupled with transcriptional profiling identified elevated GABA levels, and a simultaneous delay in *KCC2* expression, as the possible underlying reason for the increased electrophysiological activities on MEAs. GABA is a fundamentally important neurotransmitter with prenatal excitatory functions and a postnatal shift towards inhibition. This shift is realized by transcriptional up-regulation of the K^+^/Cl^−^ co-transporter *KCC2*, which actively lowers intracellular chlorine levels and thereby reverses the passive Cl^−^ transport by the GABA receptor [67]. A disruption of this pivotal shift has so far not been described for CSB patients, however, it causes developmental delays and disorders in other neurodevelopmental diseases, such as autism spectrum disorder or attention deficit hyperactivity disorder (ADHD) [67, 68]. Opposite electrophysiological findings in hiPSC-derived neuron/glia mixed cultures were published by Vessoni et al. [32]. Differences in activity might be explained by dissimilarity of patient and control cells’ genetic backgrounds, endpoint measures only at one time point, general low network activity or other aspects of the MEA protocol. Part of these limitations could be circumvented by the addition of isogenic control cell lines in our study together with a more robust baseline electrical activity. Treatment with Tubastatin A during the 7 WIV did not antagonize the elevated electrical activity suggesting a different, yet unknown, pathophysiological mode-of-action.

A third neuropathological phenotype found in CSB patients is hypomyelination [1, 7, 14, 51, 69]. Postnatal lack of myelin in offspring can be evoked by multiple causes ranging from disturbed oligodendrocyte precursor (OPC) cell proliferation, e.g. through Notch pathway inhibition [70], OPC death e.g. by increased oxidative stress or excitotoxicity [71] to inhibited oligodendrocyte maturation, e.g. by deficiency of thyroid hormone [72]. Studying hypomyelination in human in vitro models has just recently become possible with appropriate protocols becoming available [44, 73–75]. We employed hiPSC-based 3D BrainSpheres, which consist of neurons, astrocytes and oligodendrocytes [44] to investigate the hypomyelination phenotype of CSB-deficient BrainSpheres in vitro. While the quantitative ICC analyses showed no difference in the number of O4^+^ oligodendrocytes in both CSB-proficient and -deficient BrainSpheres, gene expression analyses revealed that CSB-deficient oligodendrocytes do not mature at the same pace as their respective isogenic controls. Similar to NPC migration, also the inhibited oligodendrocyte maturation can be partially rescued through HDAC-inhibition via the HDAC6-specific Tubastatin A and the pan-inhibitor SAHA. A number of studies have suggested a role of HDACs in the regulation of rodent oligodendrocyte differentiation and maturation, which renders them promising targets in different neurological pathologies [76–78]. Here we show for the first time that altered human oligodendrocyte maturation in an organotypic CSB disease model can be rescued by pharmacological intervention using HDAC inhibitors.

To date, clinically approved HDAC-inhibitors, such as SAHA (aka Vorinostat), are mainly employed as anti-cancer agents [79, 80]. Newer developments however, have brought attention to HDAC-inhibitors in other applications, for instance to treat HIV infections, muscular dystrophies, inflammatory diseases, as well as neurodegenerative diseases, such as Alzheimer’s Disease, frontotemporal dementia and Friedreich's ataxia [81]. HDACs might therefore also be promising targets for developing treatment strategies for CSB. Although the ideal time point of a potential treatment remains to be investigated, an early intervention during child development appears beneficial to prevent disease progression or even weaken the initial development. However, our data suggests that a combination of drugs might be necessary to target the different cellular adversities observed in the CSB-deficient neural models.

### Limitations of the study

In our study we used fit-for-purpose hiPSC-derived 3D in vitro models to discover and investigate multiple neurodevelopmental endophenotypes caused by CSB-deficiency. Thereby, we broadly covered multiple cellular pathomechanisms on the expense of deeply uncovering single molecular mechanisms in full detail. For example, we provided first evidence for migration defects correlated with cytoskeletal alterations such as thickened actin stress fibers. Future research should focus on lamellipodia and filopodia which will enhance our understanding of impaired actin migratory structures in CSB patients. Nevertheless, we were able to provide a first line of evidence that HDAC-dependent and -independent mechanisms converge on the pathophysiology of CSB patients. Especially the delayed GABA switch in CSB-deficient hiPSC-based neural in vitro models should be investigated in more detail, as was previously published for Rett Syndrome [82] and schizophrenia [83]. Another limitation of our study is the lack of microglia in the neural 3D models. Although not originating from neural stem cells, microglia colonize the developing brain between gestational weeks 4 and 24 [84]. They influence brain development by refining CNS formation and function, e.g. synapse formation, circuit sculpting, myelination, plasticity, and cognition. Microglia functional alterations have been associated with neurodevelopmental diseases [85, 86]. Therefore, especially for testing possible therapeutics in 3D neural models like BrainSpheres, microglia presence will further enhance the predictive value of the models.

With the different neural 3D in vitro models, we provide human-relevant multicellular systems that are already advantageous over tumor cell lines or pure neuronal cultures. However, these structures lack vascularization and, as elaborated above, immune cells. Therefore, these models have limitations when it comes to disease modelling involving inter-organ-crosstalk. Nevertheless, our study shows, that 3D in vitro brain models can provide what animal models often cannot: revealing fundamental disease mechanisms and therapeutic targets. With our work, we aim to spark further investigation into the pathophysiological mechanisms of CSB specifically, and increased usage of hiPSC-based 3D cultures for disease modelling and personalized medicine in general.

### Supplementary Information

Below is the link to the electronic supplementary material.

Supplementary file1 (DOCX 2976 KB)

Supplementary file2 (XLSX 32 KB)

## Data Availability

All data reported in this paper will be shared by the lead contact upon request. RNA Sequencing data have been deposited at GEO and are publicly available as of the date of publication. The accession number is listed in the resources table. This paper does not report original code. Any additional information required to reanalyze the data reported in this paper is available from the lead contact upon request.

## References

[1] Karikkineth AC, Scheibye-Knudsen M, Fivenson E et al (2017) Cockayne syndrome: clinical features, model systems and pathways. Ageing Res Rev 33:3–17. 10.1016/j.arr.2016.08.002 27507608 10.1016/j.arr.2016.08.002 PMC5195851

[2] Laugel V, Dalloz C, Tobias ES et al (2008) Cerebro-oculo-facio-skeletal syndrome: three additional cases with CSB mutations, new diagnostic criteria and an approach to investigation. J Med Genet 45:564–571. 10.1136/jmg.2007.057141 18628313 10.1136/jmg.2007.057141

[3] Schmickel RD, Chu EHY, Trosko JE, Chang CC (1977) Cockayne syndrome: a cellular sensitivity to ultraviolet light. Pediatrics 60:135–139 887325 10.1542/peds.60.2.135

[4] Paddison PM, Moossy J, Derbres VJ, Klopfer W (1963) Cockayne’s syndrome. A report of five new cases with biochemical, chromosomal, dermatologic, genetic and neuropathologic observations. Dermatol Trop Ecol Geogr 15:195–203 14156156

[5] Moossy J (1967) The neuropathology of Cockayne’s syndrome. J Neuropathol Exp Neurol 26:654–660. 10.1097/00005072-196710000-00010 6053735 10.1097/00005072-196710000-00010

[6] Sugarman GI, Landing BH, Reed WB (1977) Cockayne syndrome: clinical study of two patients and neuropathologic findings in one. Clin Pediatr (Phila) 16:225–232. 10.1177/000992287701600304 837626 10.1177/000992287701600304

[7] Laugel V, Dalloz C, Durand M et al (2010) Mutation update for the CSB/ERCC6 and CSA/ERCC8 genes involved in Cockayne syndrome. Hum Mutat 31:113–126. 10.1002/humu.21154 19894250 10.1002/humu.21154

[8] Kraemer KH, Patronas NJ, Schiffmann R et al (2008) Cockayne syndrome: a complex genotype-phenotype. Neuroscience 145:1388–1396 10.1016/j.neuroscience.2006.12.020 PMC2288663 17276014

[9] Chikhaoui A, Kraoua I, Calmels N et al (2022) Heterogeneous clinical features in Cockayne syndrome patients and siblings carrying the same CSA mutations. Orphanet J Rare Dis 17:121. 10.1186/s13023-022-02257-1 35248096 10.1186/s13023-022-02257-1 PMC8898519

[10] Natale V (2011) A comprehensive description of the severity groups in Cockayne syndrome. Am J Med Genet A 155A(5):1081–1095. 10.1002/ajmg.a.33933 21480477 10.1002/ajmg.a.33933

[11] De Waard H, De Wit J, Gorgels TGMF et al (2003) Cell type-specific hypersensitivity to oxidative damage in CSB and XPA mice. DNA Repair (Amst) 2:13–25. 10.1016/S1568-7864(02)00188-X 12509265 10.1016/S1568-7864(02)00188-X

[12] Gorgels TGMF, van der Pluijm I, Brandt RMC et al (2007) Retinal degeneration and ionizing radiation hypersensitivity in a mouse model for Cockayne syndrome. Mol Cell Biol 27:1433–1441. 10.1128/mcb.01037-06 17145777 10.1128/mcb.01037-06 PMC1800713

[13] Jaarsma D, van der Pluijm I, van der Horst GTJ, Hoeijmakers JHJ (2013) Cockayne syndrome pathogenesis: lessons from mouse models. Mech Ageing Dev 134:180–195. 10.1016/j.mad.2013.04.003 23591128 10.1016/j.mad.2013.04.003

[14] Vessoni AT, Guerra CCC, Kajitani GS et al (2020) Cockayne syndrome: the many challenges and approaches to understand a multifaceted disease. Genet Mol Biol. 10.1590/1678-4685-GMB-2019-0085 32453336 10.1590/1678-4685-GMB-2019-0085 PMC7250278

[15] Xu Y, Wu Z, Liu L et al (2019) Rat model of Cockayne syndrome neurological disease. Cell Rep 29:800-809.e5. 10.1016/j.celrep.2019.09.028 31644904 10.1016/j.celrep.2019.09.028

[16] Van der Horst GTJ, Van Steeg H, Berg RJW et al (1997) Defective transcription-coupled repair in Cockayne syndrome B mice is associated with skin cancer predisposition. Cell 89:425–435. 10.1016/S0092-8674(00)80223-8 9150142 10.1016/S0092-8674(00)80223-8

[17] Frouin E, Laugel V, Durand M et al (2013) Dermatologic findings in 16 patients with Cockayne syndrome and Cerebro-oculo-facial-skeletal syndrome. JAMA Dermatol 149:1414–1418. 10.1001/jamadermatol.2013.6683 24154677 10.1001/jamadermatol.2013.6683

[18] Majora M, Sondenheimer K, Knechten M et al (2018) HDAC inhibition improves autophagic and lysosomal function to prevent loss of subcutaneous fat in a mouse model of Cockayne syndrome. Sci Transl Med. 10.1126/scitranslmed.aam7510 30158153 10.1126/scitranslmed.aam7510

[19] Fritsche E, Tigges J, Hartmann J et al (2020) Neural in vitro models for studying substances acting on the central nervous system. Handb Exp Pharmacol. 10.1007/164_2020_367 10.1007/164_2020_367 32594299

[20] Fritsche E, Haarmann-Stemmann T, Kapr J et al (2020) Stem cells for next level toxicity testing in the 21st century. Small 2006252:1–31. 10.1002/smll.202006252 10.1002/smll.202006252 33354870

[21] Azar J, Bahmad HF, Daher D et al (2021) The use of stem cell-derived organoids in disease modeling: an update. Int J Mol Sci. 10.3390/ijms22147667 34299287 10.3390/ijms22147667 PMC8303386

[22] Pamies D, Wiersma D, Katt ME et al (2022) Human organotypic brain model as a tool to study chemical-induced dopaminergic neuronal toxicity. Neurobiol Dis 169:105719. 10.1016/j.nbd.2022.105719 35398340 10.1016/j.nbd.2022.105719 PMC9298686

[23] Park J, Wetzel I, Marriott I et al (2018) A 3D human triculture system modeling neurodegeneration and neuroinflammation in Alzheimer’s disease. Nat Neurosci 21:941–951. 10.1038/s41593-018-0175-4 29950669 10.1038/s41593-018-0175-4 PMC6800152

[24] Martins S, Hacheney I, Teichweyde N et al (2021) Generation of an induced pluripotent stem cell line (IUFi001) from a Cockayne syndrome patient carrying a mutation in the ERCC6 gene. Stem Cell Res. 10.1016/j.scr.2021.102456 34271225 10.1016/j.scr.2021.102456

[25] Pasca AM, Sloan SA, Clarke LE et al (2015) Functional cortical neurons and astrocytes from human pluripotent stem cells in 3D culture. Nat Methods 12:671–678. 10.1038/nmeth.3415 26005811 10.1038/nmeth.3415 PMC4489980

[26] Mayne L, Lehmann AR (1982) Failure of RNA synthesis to recover after UV irradiation: an early defect in cells from individuals with Cockayne syndrome and xeroderma pigmentosum. Mutat Res 96:140. 10.1016/0027-5107(82)90047-1 10.1016/0027-5107(82)90047-1 6174225

[27] Troelstra C, van Gool A, de Wit J et al (1992) ERCC6, a member of a subfamily of putative helicases, is involved in Cockayne’s syndrome and preferential repair of active genes. Cell 71:939–953. 10.1016/0092-8674(92)90390-X 1339317 10.1016/0092-8674(92)90390-X

[28] Rapin I, Lindenbaum Y, Dickson DW et al (2000) Cockayne syndrome and xeroderma pigmentosum: DNA repair disorders with overlaps and paradoxes. Neurology 55:1442–1449. 10.1212/WNL.55.10.1442 11185579 10.1212/WNL.55.10.1442 PMC4459578

[29] Lindenbaum Y, Dickson D, Rosenbaum P et al (2001) Xeroderma pigmentosum/Cockayne syndrome complex: first neuropathological study and review of eight other cases. Eur J Paediatr Neurol 5:225–242. 10.1053/ejpn.2001.0523 11764181 10.1053/ejpn.2001.0523

[30] Ciaffardini F, Nicolai S, Caputo M et al (2014) The Cockayne syndrome B protein is essential for neuronal differentiation and neuritogenesis. Cell Death Dis 5:e1268–e1311. 10.1038/cddis.2014.228 24874740 10.1038/cddis.2014.228 PMC4047889

[31] Wang Y, Jones-Tabah J, Chakravarty P et al (2016) Pharmacological bypass of Cockayne syndrome B function in neuronal differentiation. Cell Rep 14:2554–2561. 10.1016/j.celrep.2016.02.051 26972010 10.1016/j.celrep.2016.02.051 PMC4806223

[32] Vessoni AT, Herai RH, Karpiak JV et al (2016) Cockayne syndrome-derived neurons display reduced synapse density and altered neural network synchrony. Hum Mol Genet 25:1271–1280. 10.1093/hmg/ddw008 26755826 10.1093/hmg/ddw008 PMC4787902

[33] Liang F, Li B, Xu Y et al (2023) Identification and characterization of necdin as a target for the Cockayne syndrome B protein in promoting neuronal differentiation and maintenance. Pharmacol Res 187:106637. 10.1016/j.phrs.2022.106637 36586641 10.1016/j.phrs.2022.106637

[34] Szepanowski L-P, Wruck W, Kapr J et al (2024) Cockayne syndrome patient iPSC-derived brain organoids and neurospheres show early transcriptional dysregulation of biological processes associated with brain development and metabolism. Cells 13(7):591. 10.3390/cells13070591 38607030 10.3390/cells13070591 PMC11011893

[35] Brunner JW, Lammertse HCA, van Berkel AA et al (2023) Power and optimal study design in iPSC-based brain disease modelling. Mol Psychiatry 28:1545–1556. 10.1038/s41380-022-01866-3 36385170 10.1038/s41380-022-01866-3 PMC10208961

[36] Hofrichter M, Nimtz L, Tigges J et al (2017) Comparative performance analysis of human iPSC-derived and primary neural progenitor cells (NPC) grown as neurospheres in vitro. Stem Cell Res 25:72–82. 10.1016/j.scr.2017.10.013 29112887 10.1016/j.scr.2017.10.013

[37] Nimtz L, Hartmann J, Tigges J et al (2020) Characterization and application of electrically active neuronal networks established from human induced pluripotent stem cell-derived neural progenitor cells for neurotoxicity evaluation. Stem Cell Res 45:101761. 10.1016/j.scr.2020.101761 32244191 10.1016/j.scr.2020.101761

[38] Pamies D, Chesnut M, Smirnova L et al (2021) Human 3D iPSC-derived brain model to study chemical-induced myelin disruption. Glia 69:E392–E394

[39] Hartmann J, Henschel N, Bartmann K et al (2023) Molecular and functional characterization of different brainsphere models for use in neurotoxicity testing on microelectrode arrays. Cells. 10.3390/cells12091270 37174670 10.3390/cells12091270 PMC10177384

[40] Hofrichter M (2016) Establishment of a hiPSC-based in vitro model to study environmental and genetic disturbances of neurodevelopmental processes. PhD Diss. Heinrich-Heine-University Duesseldorf. urn:nbn:de:hbz:061-20170424-091020-5

[41] Ramachandran H, Martins S, Kontarakis Z et al (2021) Fast but not furious: a streamlined selection method for genome-edited cells. Life Sci Alliance. 10.26508/lsa.202101051 33903218 10.26508/lsa.202101051 PMC8127327

[42] Nguyen T, Ramachandran H, Martins S et al (2022) Identification of genome edited cells using CRISPRnano. Nucleic Acids Res 50:W199–W203. 10.1093/nar/gkac440 35640601 10.1093/nar/gkac440 PMC9252781

[43] Tigges J, Bielec K, Brockerhoff G et al (2021) Academic application of good cell culture practice for induced pluripotent stem cells. Altex. 10.14573/altex.2101221 33963415 10.14573/altex.2101221

[44] Pamies D, Barreras P, Block K et al (2017) A human brain microphysiological system derived from induced pluripotent stem cells to study neurological diseases and toxicity. Altex 34:362–376. 10.14573/altex.1609122 27883356 10.14573/altex.1609122 PMC6047513

[45] Baumann J, Gassmann K, Masjosthusmann S et al (2016) Comparative human and rat neurospheres reveal species differences in chemical effects on neurodevelopmental key events. Arch Toxicol 90:1415–1427. 10.1007/s00204-015-1568-8 26216354 10.1007/s00204-015-1568-8

[46] Kong L, Zhang Y, Ye ZQ et al (2007) CPC: assess the protein-coding potential of transcripts using sequence features and support vector machine. Nucleic Acids Res. 10.1093/nar/gkm391 17631615 10.1093/nar/gkm391 PMC1933232

[47] Gu J, Weber K, Klemp E et al (2012) Identifying core features of adaptive metabolic mechanisms for chronic heat stress attenuation contributing to systems robustness. Integr Biol 4:480–493. 10.1039/c2ib00109h 10.1039/c2ib00109h 22402787

[48] Shim SH, Lee SK, Lee DW et al (2020) Loss of function of rice plastidic glycolate/glycerate translocator 1 impairs photorespiration and plant growth. Front Plant Sci. 10.3389/fpls.2019.01726 32038690 10.3389/fpls.2019.01726 PMC6993116

[49] Poirier K, Lebrun N, Broix L et al (2013) Mutations in TUBG1, DYNC1H1, KIF5C and KIF2A cause malformations of cortical development and microcephaly. Nat Genet 45:639–647. 10.1038/ng.2613 23603762 10.1038/ng.2613 PMC3826256

[50] Becerra-Solano LE, Mateos-Sánchez L, López-Muñoz E (2021) Microcephaly, an etiopathogenic vision. Pediatr Neonatol 62:354–360. 10.1016/j.pedneo.2021.05.008 34112604 10.1016/j.pedneo.2021.05.008

[51] Laugel V, Dalloz C, Stary A et al (2008) Deletion of 5′ sequences of the CSB gene provides insight into the pathophysiology of Cockayne syndrome. Eur J Hum Genet 16:320–327. 10.1038/sj.ejhg.5201991 18183039 10.1038/sj.ejhg.5201991

[52] Bartmann K, Bendt F, Dönmez A et al (2023) A human iPSC-based in vitro neural network formation assay to investigate neurodevelopmental toxicity of pesticides. bioRxiv. 10.1101/2023.01.12.523741 10.1101/2023.01.12.523741 37158368

[53] Ben-Ari Y (2002) Excitatory actions of GABA during development: the nature of the nurture. Nat Rev Neurosci 3:728–739. 10.1038/nrn920 12209121 10.1038/nrn920

[54] de Lange ECM, Hammarlund-Udenaes M (2015) Translational aspects of blood-brain barrier transport and central nervous system effects of drugs: from discovery to patients. Clin Pharmacol Ther 97:380–394. 10.1002/CPT.76 25670219 10.1002/CPT.76

[55] Arrowsmith J, Miller P (2013) Trial watch: phase II and phase III attrition rates 2011–2012. Nat Rev Drug Discov 12:569. 10.1038/nrd4090 23903212 10.1038/nrd4090

[56] Cummings JL, Morstorf T, Zhong K (2014) Alzheimer’s disease drug-development pipeline: few candidates, frequent failures. Alzheimer’s Res Ther. 10.1186/alzrt269 10.1186/alzrt269 PMC4095696 25024750

[57] Ashmawi NS, Hammoda MA (2022) Early prediction and evaluation of risk of autism spectrum disorders. Cureus. 10.7759/cureus.23465 35481307 10.7759/cureus.23465 PMC9034898

[58] Kenific CM, Wittmann T, Debnath J (2016) Autophagy in adhesion and migration. J Cell Sci 129:3685–3693. 10.1242/jcs.188490 27672021 10.1242/jcs.188490 PMC5087656

[59] Kenific CM, Stehbens SJ, Goldsmith J et al (2016) NBR 1 enables autophagy-dependent focal adhesion turnover. J Cell Biol 212:577–590. 10.1083/jcb.201503075 26903539 10.1083/jcb.201503075 PMC4772495

[60] Hernandez SJ, Fote G, Reyes-Ortiz AM et al (2021) Cooperation of cell adhesion and autophagy in the brain: functional roles in development and neurodegenerative disease. Matrix Biol Plus 12:100089. 10.1016/j.mbplus.2021.100089 34786551 10.1016/j.mbplus.2021.100089 PMC8579148

[61] Clawson GA (2016) Histone deacetylase inhibitors as cancer therapeutics. Ann Transl Med. 10.21037/atm.2016.07.22 27568481 10.21037/atm.2016.07.22 PMC4980376

[62] Hartlaub AM, McElroy CA, Maitre NL, Hester ME (2019) Modeling human brain circuitry using pluripotent stem cell platforms. Front Pediatr 7:1–8. 10.3389/fped.2019.00057 30891437 10.3389/fped.2019.00057 PMC6411708

[63] Pelkonen A, Pistono C, Klecki P et al (2022) Functional characterization of human pluripotent stem cell-derived models of the brain with microelectrode arrays. Cells. 10.3390/cells11010106 10.3390/cells11010106 PMC8750870 35011667

[64] Silbereis JC, Pochareddy S, Zhu Y et al (2016) The cellular and molecular landscapes of the developing human central nervous system. Neuron 89:248. 10.1016/j.neuron.2015.12.008 26796689 10.1016/j.neuron.2015.12.008 PMC4959909

[65] Budday S, Steinmann P, Kuhl E (2015) Physical biology of human brain development. Front Cell Neurosci 9:1–17. 10.3389/fncel.2015.00257 26217183 10.3389/fncel.2015.00257 PMC4495345

[66] Vasudevan P, Suri M (2017) A clinical approach to developmental delay and intellectual disability. Clin Med J R Coll Physicians Lond 17:558–561. 10.7861/clinmedicine.17-6-558 10.7861/clinmedicine.17-6-558 PMC6297696 29196358

[67] Peerboom C, Wierenga CJ (2021) The postnatal GABA shift: a developmental perspective. Neurosci Biobehav Rev 124:179–192. 10.1016/j.neubiorev.2021.01.024 33549742 10.1016/j.neubiorev.2021.01.024

[68] Pozzi D, Rasile M, Corradini I, Matteoli M (2020) Environmental regulation of the chloride transporter KCC2: switching inflammation off to switch the GABA on? Transl Psychiatry. 10.1038/s41398-020-01027-6 33060559 10.1038/s41398-020-01027-6 PMC7562743

[69] Gitiaux C, Blin-Rochemaure N, Hully M et al (2015) Progressive demyelinating neuropathy correlates with clinical severity in Cockayne syndrome. Clin Neurophysiol 126:1435–1439. 10.1016/j.clinph.2014.10.014 25453614 10.1016/j.clinph.2014.10.014

[70] Ying Y-Q, Yan X-Q, Jin S-J et al (2018) Inhibitory effect of LPS on the proliferation of oligodendrocyte precursor cells through the notch signaling pathway in intrauterine infection-induced rats. Curr Med Sci 38:840–846. 10.1007/s11596-018-1951-9 30341518 10.1007/s11596-018-1951-9

[71] Volpe JJ, Kinney HC, Jensen FE, Rosenberg PA (2011) The developing oligodendrocyte: key cellular target in brain injury in the premature infant. Int J Dev Neurosci 29:423–440. 10.1016/j.ijdevneu.2011.02.012 21382469 10.1016/j.ijdevneu.2011.02.012 PMC3099053

[72] Dach K, Bendt F, Huebenthal U et al (2017) BDE-99 impairs differentiation of human and mouse NPCs into the oligodendroglial lineage by species-specific modes of action. Sci Rep. 10.1038/srep44861 28317842 10.1038/srep44861 PMC5357893

[73] Ehrlich M, Mozafari S, Glatza M et al (2017) Rapid and efficient generation of oligodendrocytes from human induced pluripotent stem cells using transcription factors. Proc Natl Acad Sci USA 114:E2243–E2252. 10.1073/pnas.1614412114 28246330 10.1073/pnas.1614412114 PMC5358375

[74] Chesnut M, Hartung T, Hogberg H, Pamies D (2021) Human oligodendrocytes and myelin in vitro to evaluate developmental neurotoxicity. Int J Mol Sci 22:7929. 10.3390/ijms22157929 34360696 10.3390/ijms22157929 PMC8347131

[75] Douvaras P, Fossati V (2015) Generation and isolation of oligodendrocyte progenitor cells from human pluripotent stem cells. Nat Protoc 10:1143–1154. 10.1038/nprot.2015.075 26134954 10.1038/nprot.2015.075

[76] Noack M, Leyk J, Richter-Landsberg C (2014) HDAC6 inhibition results in tau acetylation and modulates tau phosphorylation and degradation in oligodendrocytes. Glia 62:535–547. 10.1002/glia.22624 24464872 10.1002/glia.22624

[77] Liu B, Chen X, Wang ZQ, Tong WM (2014) Nbn gene inactivation in the CNS of mouse inhibits the myelinating ability of the mature cortical oligodendrocytes. Glia 62:133–144. 10.1002/glia.22593 24272708 10.1002/glia.22593

[78] Tauheed AM, Ayo JO, Kawu MU (2016) Regulation of oligodendrocyte differentiation: insights and approaches for the management of neurodegenerative disease. Pathophysiology 23:203–210. 10.1016/j.pathophys.2016.05.007 27342760 10.1016/j.pathophys.2016.05.007

[79] Yadav R, Mishra P, Yadav D (2019) Histone deacetylase inhibitors: a prospect in drug discovery. Turk J Pharm Sci 16:101–114. 10.4274/tjps.75047 32454703 10.4274/tjps.75047 PMC7227979

[80] Smalley JP, Cowley SM, Hodgkinson JT (2020) Bifunctional HDAC therapeutics: one drug to rule them all? Molecules. 10.3390/molecules25194394 32987782 10.3390/molecules25194394 PMC7583022

[81] Bondarev AD, Attwood MM, Jonsson J et al (2021) Recent developments of HDAC inhibitors: emerging indications and novel molecules. Br J Clin Pharmacol 87:4577–4597. 10.1111/bcp.14889 33971031 10.1111/bcp.14889

[82] Tang X, Kim J, Zhou L et al (2016) KCC2 rescues functional deficits in human neurons derived from patients with Rett syndrome. Proc Natl Acad Sci USA 113:751–756. 10.1073/pnas.1524013113 26733678 10.1073/pnas.1524013113 PMC4725523

[83] Toritsuka M, Yoshino H, Makinodan M et al (2021) Developmental dysregulation of excitatory-to-inhibitory GABA-polarity switch may underlie schizophrenia pathology: a monozygotic-twin discordant case analysis in human iPS cell-derived neurons. Neurochem Int. 10.1016/j.neuint.2021.105179 34500023 10.1016/j.neuint.2021.105179

[84] Menassa DA, Gomez-Nicola D (2018) Microglial dynamics during human brain development. Front Immunol. 10.3389/fimmu.2018.01014 29881376 10.3389/fimmu.2018.01014 PMC5976733

[85] Allen NJ, Lyons DA (2018) Glia as architects of central nervous system formation and function. Science 185:181–185 10.1126/science.aat0473 PMC6292669 30309945

[86] Bar E, Barak B (2019) Microglia roles in synaptic plasticity and myelination in homeostatic conditions and neurodevelopmental disorders. Glia 67:2125–2141. 10.1002/glia.23637 31058364 10.1002/glia.23637

[87] Tau GZ, Peterson BS (2010) Normal development of brain circuits. Neuropsychopharmacology 35:147–168. 10.1038/npp.2009.115 19794405 10.1038/npp.2009.115 PMC3055433

