# Supplementary material for: HiPSC-derived 3D neural models reveal neurodevelopmental pathomechanisms of the Cockayne Syndrome B
Source: Cell Mol Life Sci. 2024 Aug 23;81(1):368. doi: 10.1007/s00018-024-05406-w (PMC11343962; doi:10.1007/s00018-024-05406-w)
Supplement: Supplementary file 1 — Supplementary file1 (DOCX 2976 KB) [file 18_2024_5406_MOESM1_ESM.docx]

HiPSC-derived 3D neural models reveal neurodevelopmental pathomechanisms of the Cockayne Syndrome B

Julia Kapr^1^, Ilka Scharkin^1^, Haribaskar Ramachandran^1^, Philipp Westhoff^2^, Marius Pollet^1^, Selina Dangeleit^1^, Gabriele Brockerhoff^1^, Andrea Rossi^1^, Katharina Koch^1,4^, Jean Krutmann^1,3^, Ellen Fritsche^1,3,4*^

^1^IUF-Leibniz Research Institute for Environmental Medicine, 40225 Düsseldorf, Germany

^2^CEPLAS Metabolism and Metabolomics Laboratory, Cluster of Excellence on Plant Science (CEPLAS), Heinrich Heine University Düsseldorf, 40225 Düsseldorf, Germany

^3^Medical Faculty, University of Düsseldorf, 40225 Düsseldorf, Germany

^4^DNTOX GmbH, 40223 Düsseldorf

^*^Corresponding author: ellen.fritsche@iuf-duesseldorf.de, +49 211 3389 217

**Supplemental Information**

**Figures**


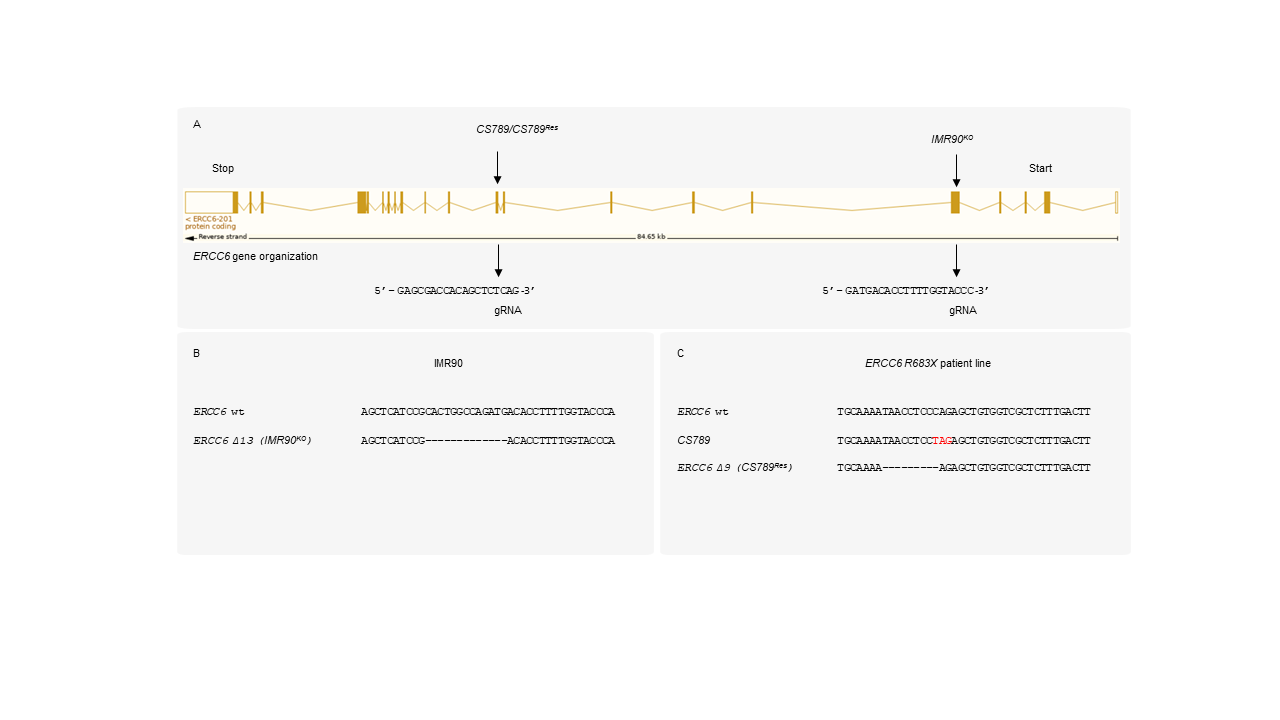


**SI Figure S1. Cell Line Information – related to ‘Two hiPSC models for neurodevelopmental key event analyses in the Cockayne Syndrome B’.** (A) Schematic view of ERCC6 gene structure modified from Ensembl (ENT00000355832). Top, ERCC6 iPSC mutants (CS789^Res^, CS789 and IMR90^KO^) carry mutations in exons pointed to by the arrows. IMR90^KO^ ERCC6 mutant was generated using commercially available iPSCs (IMR90^WT^). CS789^Res^ mutant was generated using the CS789 patient-derived iPSC line. Bottom, arrows point to the gRNA sequences used to generate ERCC6 mutants (CS789 and IMR90^KO^). (B) Next generation sequencing alignment of IMR90^WT^ and IMR90^KO^ mutant. Depicted is a deletion of 13 bp in the IMR90^KO^ mutant. (C) Next generation sequencing alignment of IMR90^WT^, CS789 (the stop codon is depicted in red), and CS789^Res^ mutant that carries 9 bp in-frame deletion that removes the premature stop codon present in the CS789 patient line.


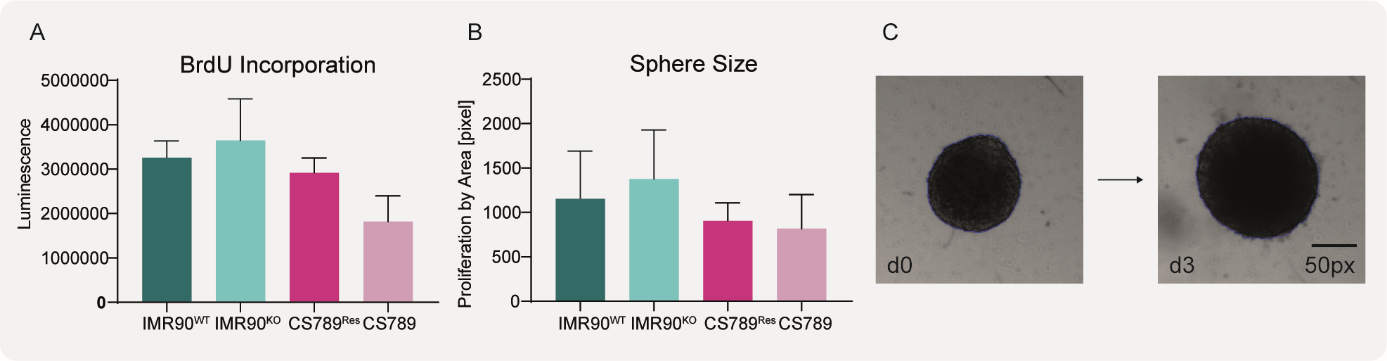


**SI Figure S2. CSB-deficient cell lines do not show altered proliferation – related to ‘CSB-deficiency is associated with inhibited neural progenitor cell migration and alterations in focal adhesion and autophagy’.** Proliferation of all cell lines was assessed in hiNPCs using the BrdU assay and area measurement. Spheres were cultivated in proliferation medium for 3 days. (A) The BrdU incorporation on day 3. The graph depicts the luminescence of each cell line. (B) The area was measured every day, and the increase in Area is depicted for each cell line. (C) Exemplary images of sphere growth from d0 to d3. N=3 biological replicates with n=3-5 spheres. All graphs depict the mean ± SEM. Abbreviations: hiNPC, human induced neural progenitor cell; BrdU, bromodeoxyuridine / 5-bromo-2'-deoxyuridine.


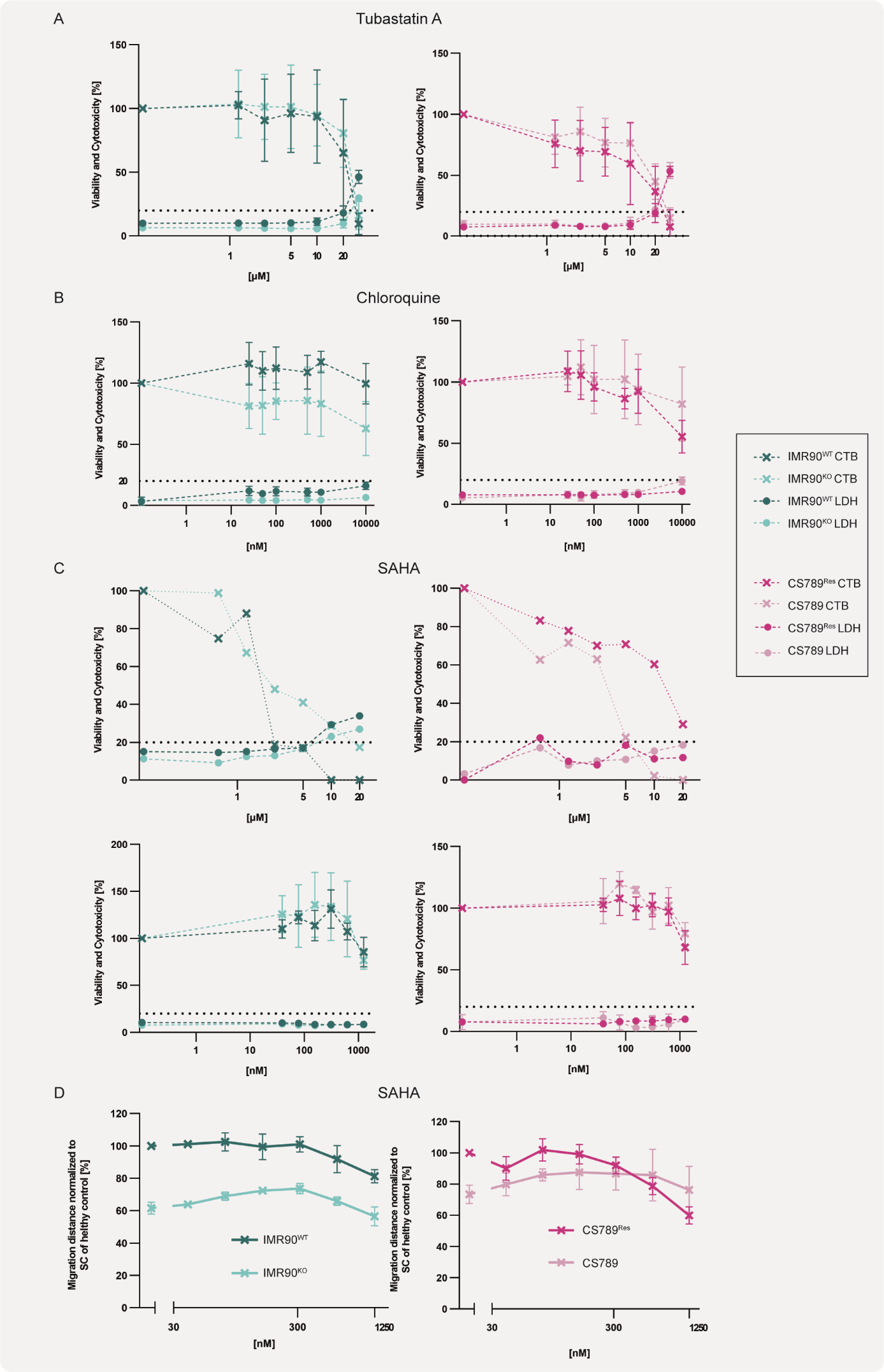


**SI Figure S3. Migration treatment supplemental information – related to ‘The HDAC6-inhibitor Tubastatin A partially rescues the migration phenotype of disease hiNPC neurospheres’.** Cytotoxicity (LDH) and viability (CTB) were measured for all Tubastatin A (A), Chloroquine (B) and SAHA (C) treatments. (C) The top two graphs depict an N=1 biological replicate with n=3-5 spheres. All other Graphs depict N=3 biological replicates with n=3-5 spheres. (D) Migration in differentiation BrainSpheres treated with different SAHA concentrations. N=3 biological replicates with n=3-5 spheres. All graphs depict the mean ± SEM.


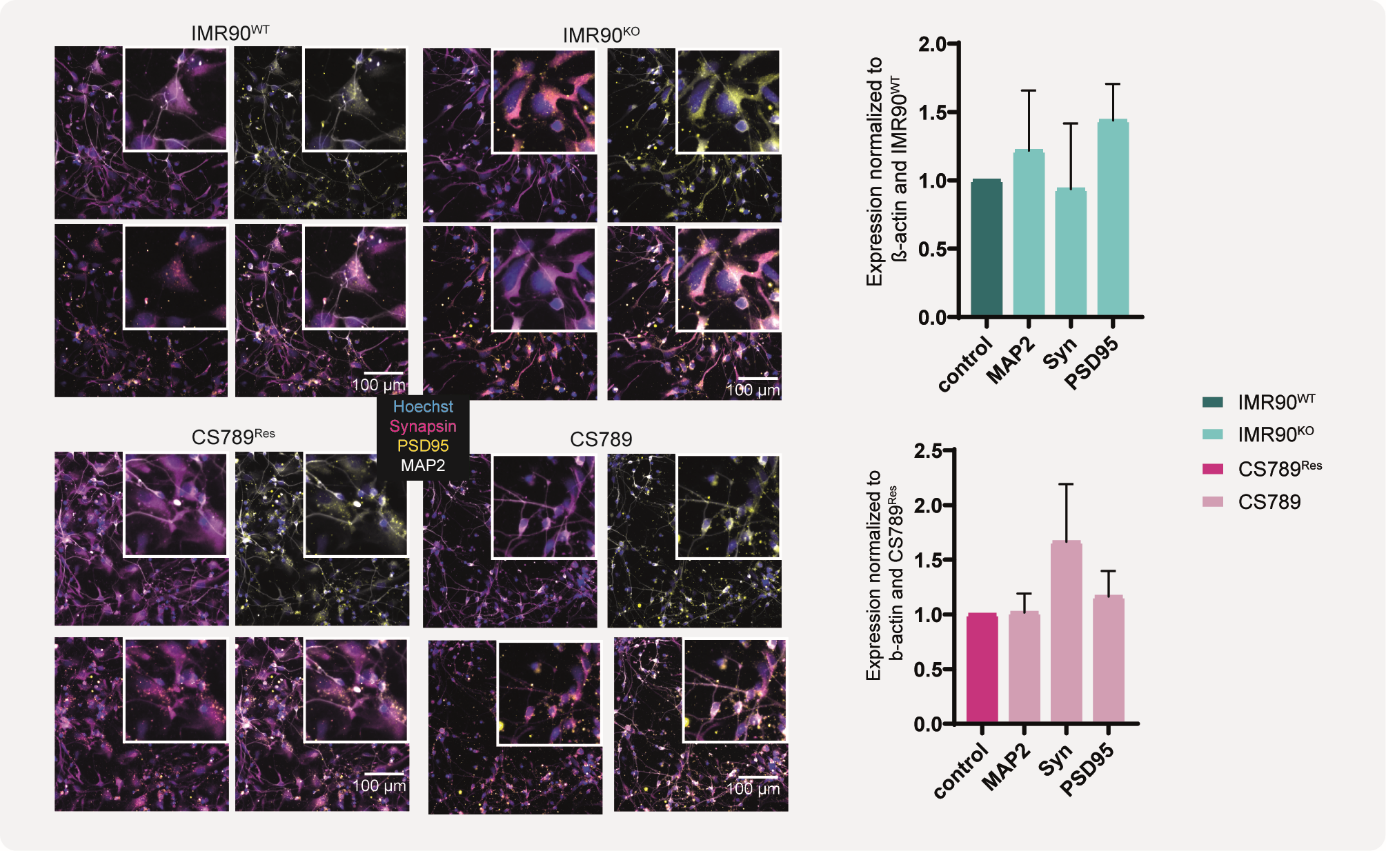


**SI Figure S4. Neuronal and synaptic markers – related to ‘Microelectrode Array measurements reveal altered neural network activity in CSB-deficient networks’.** Left: ICC stainings of plated hiNPC spheres after 3 DIV. Representative images of each cell line are depicted. Right: qPCR analyses of MAP2 (neuronal marker), SYN (pre-synaptic marker) and PSD95 (post-synaptic marker) after 3 DIV, normalized to the housekeeper ß-acting and the respective control cell line. Graps depict mean±SEM. Abbreviations: ICC, immunocytochemistry; hiNPC, human induced neural progenitor cell; DIV, days in vitro.


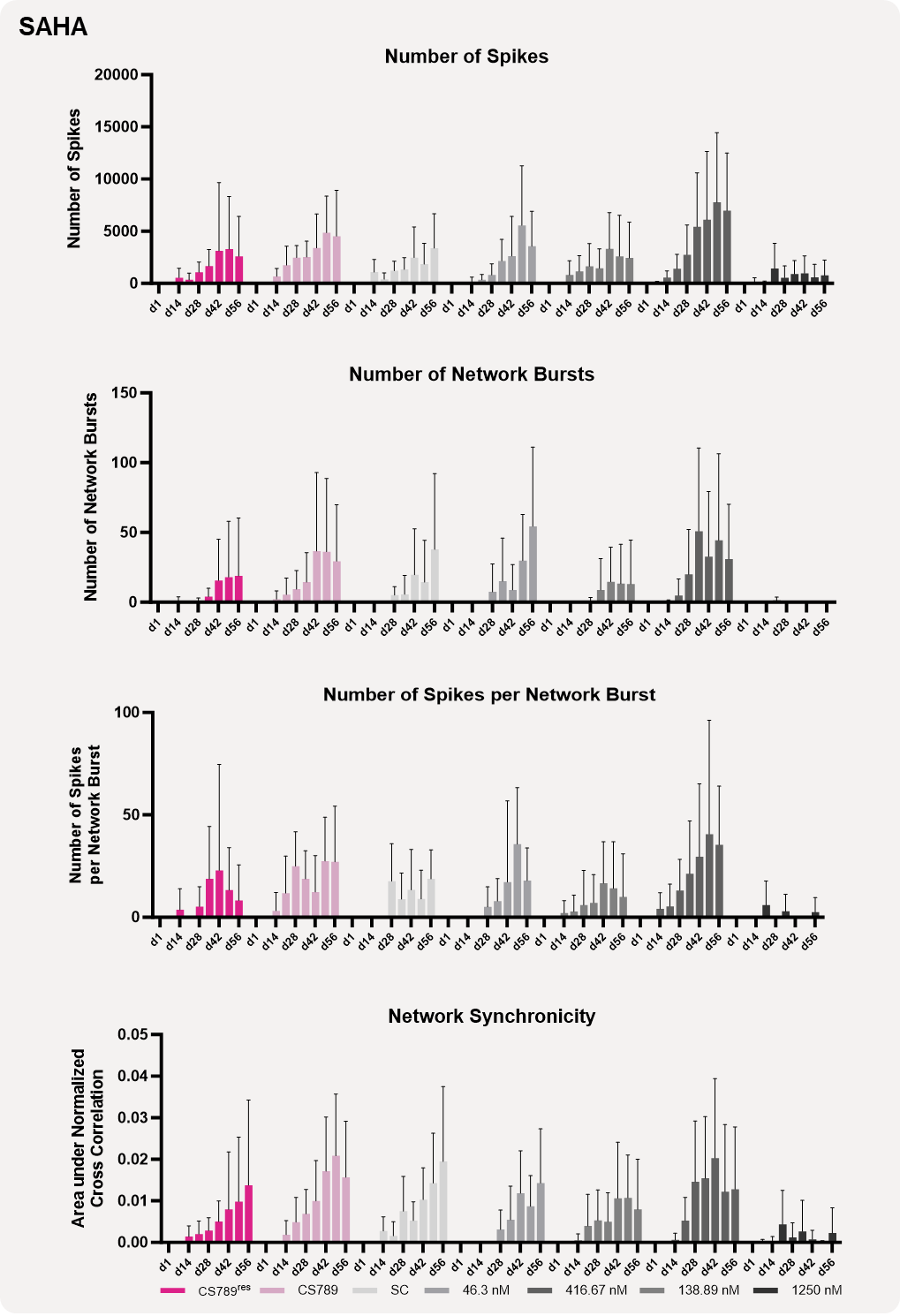

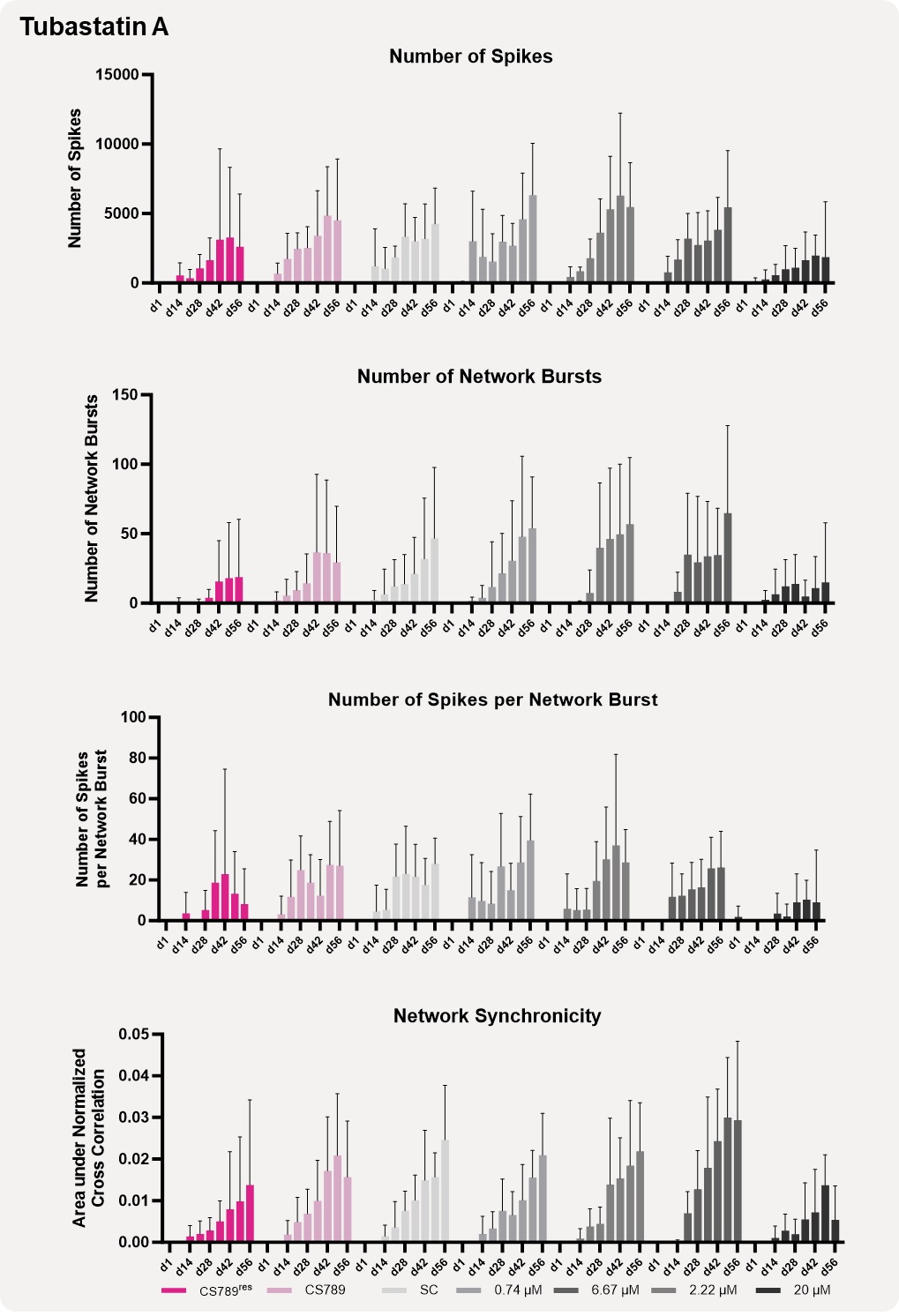


**SI Figure S5. Rescue trials with HDAC-inhibitors TubA and SAHA did not change neural network activities in disease cell lines. – Related to ‘Microelectrode Array measurements reveal altered neural network activity in CSB-deficient networks’.** The electrical activity of neural networks over time under exposure of increasing concentrations to HDAC inhibitors Tubastatin A (left) and SAHA (right) is depicted and compared to the respective control. The graphs show the mean ± SD for the selected parameters number of spikes, number of network bursts, area under normalized cross correlation (network synchrony) and number of spikes per network burst (N= 8-12 wells with n=64-96 electrodes were evaluated). Each time point comprises a 15 min measurement.


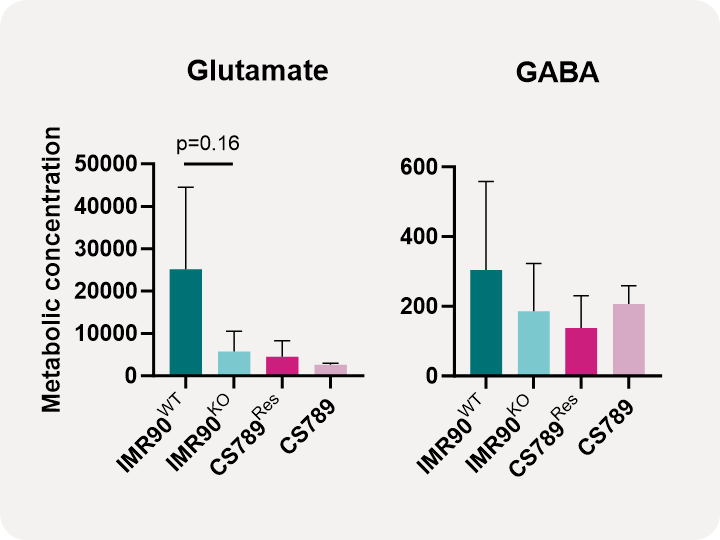


**SI Figure S6. Neurotransmitter Metabolomics. – Related to ‘Altered GABA levels and KCC2 expression hint towards a delayed GABA switch in disease cell lines’.** HiNPC neurospheres were plated and differentiated for 14 DIV, before GC-MS was performed. Graphs depict the ratios of the relative metabolite concentrations of neurotransmitters glutamate and GABA (N=3 biological replicates, mean ± SEM).


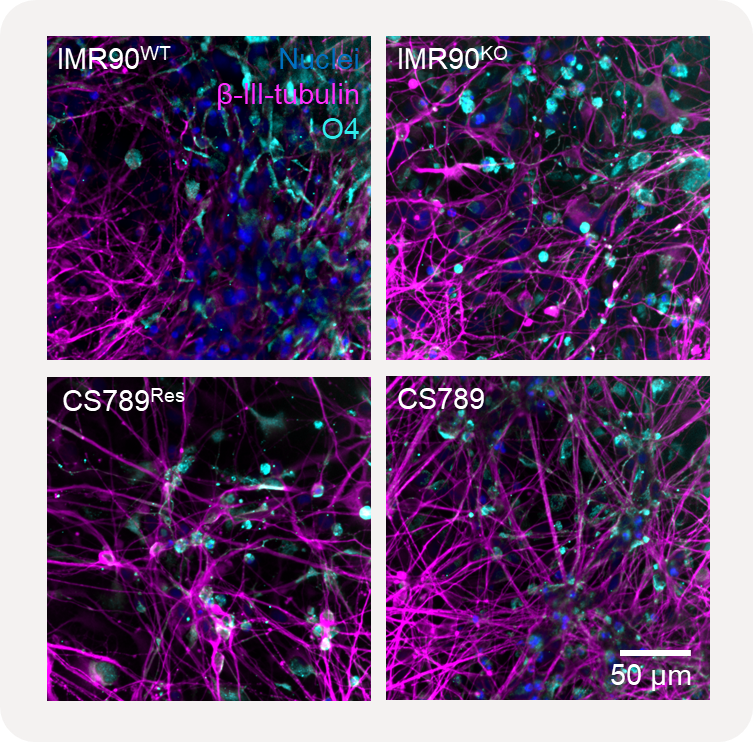


**SI Figure S7. Quantification of O4-positive cells after 4 weeks of differentiation. – Related to ‘CSB-deficiency leads to hindered oligodendrocyte maturation’.** Representative images of 2D cultures used for quantification of O4 positive cells after 4 weeks of adherent oligodendrocyte differentiation. β-III-tubulin – neuronal marker, O4 – oligodendrocyte marker, Hoechst – nuclei.


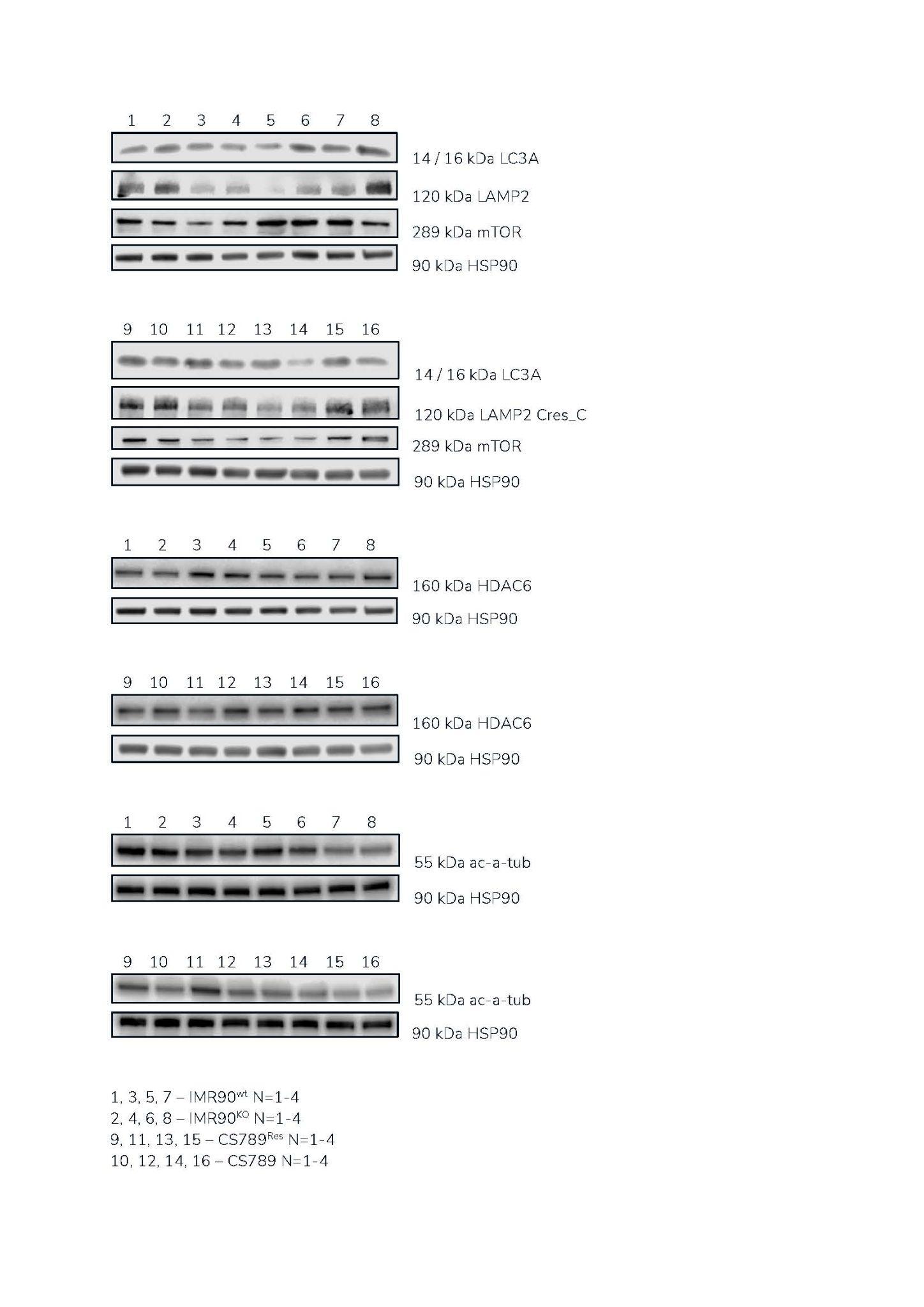


**SI Figure S8. Western Blots.** 1, 3, 5 and 7 represent IMR90wt N=1-4. 2, 4, 6 and 8 represent IMR90KO N=1-4. 9, 11, 13 and 15 represent CS789Res N=1-4. 10, 12, 14 and 16 represent CS789 N=1-4.

**Tables**

**SI Table S1. CS789 – Patient information**^1^ **– related to ‘Two hiPSC models for neurodevelopmental key event analyses in the Cockayne Syndrome B’**.

| Patient ID | CS789VI |
| --- | --- |
| Origin | UK |
| Mutation | Point mutation (2047C>T; p.Arg683x) |
| Donor Cell Type | Dermal fibroblasts, male |
| Clinical Classification | Cerebro-oculofacio-skeletal syndrome (COFS) |
| Growth failure | + |
| Low birth weight | + |
| Cachexia | - |
| Intellectual Disability | Severe |
| Microcephaly | Congenital |
| Seizures | + |
| Cataracts | Congenital |
| Microphthalmia | + |
| Retinal degradation | - |
| Deafness | + |
| Clinical photosensitivity | + |
| Dental anomalies | - |
| Arthrogryposis | + |
| Age at Death | 10 months |

**SI Table S2. qPCR Primers.** All primers supplied by Eurofins.

| ß-actin | FW | CAG GAA GTC CCT TGC CAT CC | NM_001101 |
| --- | --- | --- | --- |
|  | RV | ACC AAA AGC CTT CAT ACA TCT CA |  |
| NKCC1 | FW | ACA AAG TTG AGG AAG AGG ATG GC | NM_001046.3 |
|  | RV | CCT GAT CTG CCG GTA TGT CTT GG |  |
| KCC2 | FW | CTA CAG CGA ACG AGA GAG CG | NM_001134771.2 |
|  | RV | CCA TCT CCT CCT CAA ACA AGG C |  |
| FABP7 | FW | TCA TCA GGA CTC TCA GCA CA | NM_001446.5 |
|  | RV | GAA CAG CAA CCA CAT CAC CA |  |
| NG2 | FW | CGG ACA CTT CTT CCG AGT GA | NM_001897.5 |
|  | RV | TAT TCC CAG CGT AGA CCT CTG |  |
| PLP | FW | TTG GCG ACT ACA AGA CCA CC | NM_000533.5 |
|  | RV | GGG AAG GCA ATA GAC TGG CA |  |
| PDGFRa | FW | ATT AAG CCG GTC CCA ACC TG | NM_006206.6 |
|  | RV | AGC TCC GTG TGC TTT CAT CA |  |
| CNPase | FW | ACT CAG GCA TCA TTC CAC CA | NM_033133.5 |
|  | RV | TGT CAA GCG TGG TGT TCA AG |  |
| Olig2 | FW | CCG ATG ACC TTT TTC TGC CG | NM_005806.4 |
|  | RV | CCA CTG CCT CCT AGC TTG TC |  |
| MBP | FW | CAG AGC GTC CGA CTA TAA ATC G | NM_001025081.2 |
|  | RV | GGT GGG TTT TCA GCG TCT A |  |

**SI Table S3. Resource Table.**

| REAGENT or RESOURCE | SOURCE | IDENTIFIER |
| --- | --- | --- |
| Antibodies | | |
| Anti-beta III tubulin conjugated to Alexa Fluor 647 | Abcam | ab190575 |
| Anti-MAP2 | Thermo Fisher | 13-1500; RRID:AB_2533001 |
| Anti-AQP4 | Merck | HPA014784;  RRID:AB_1844967 |
| Anti-LAMP2 | Santa Cruz | sc-18822;  RRID:AB_626858 |
| Anti-O4 | R&D Systems | MAB1326;  RRID:AB_357617 |
| Anti-CSB | GeneTex | GTX104589 |
| Anti-LC3A | Abcam | ab52768;  RRID:AB_881226 |
| Anti-mTOR | Cell Signaling | mAb2983 |
| Anti-acetyl-alpha-tubulin | Cell Signaling | mAb5335 |
| Anti-HADC6 | Cell Signaling | mAb7558 |
| Anti-HSP90 | Cell Signaling | mAb4877 |
| Hoechst 34580 | Thermo Fisher | H21486 |
| Alexa Fluor 488 Phalloidin | Invitrogen | A12379 |
| Anti-mouse Alexa Fluor 488 | Invitrogen | A11001;  RRID:AB_2534069 |
| Anti-mouse Alexa Fluor 546 | Invitrogen | A11030;  RRID:AB_2534089 |
| Anti-rabbit Alexa Fluor 546 | Invitrogen | A11010;  RRID:AB_2534077 |
| Anti-mouse Alexa Fluor 488 | Invitrogen | A-21042;  RRID:AB_2535711 |
| Anti-mouse | LI-COR Bioscience | 926-80010 |
| Anti-rabbit | LI-COR Biosciences | 926-80011 |
| Chemicals, peptides, and recombinant proteins | | |
| Laminin521 | Biolamina | LN521-05 |
| Laminin111 | Merck | L2020 |
| EDTA | Thermo Fisher | 15575020 |
| ROCK inhibitor | Hello Bio | HB2297 |
| Penicillin/Streptomycin | PAN-Biotech | P06-07100 |
| Human recombinant fibroblast growth factor (hFGF) | R&D Systems | 233-FB |
| mTeSR1 | StemCell Technologies | 5850 |
| mTeSR1 supplement | StemCell Technologies | 5850 |
| Penicillin/Streptomycin | PAN-Biotech | P06-07100 |
| iPS-brew XF, human | StemMACS | 130-104-368 |
| iPS Brew XF supplement | StemMACS | 130-104-368 |
| Penicillin/Streptomycin | PAN-Biotech | P06-07100 |
| Knockout Serum Replacement | Invitrogen | 10828028 |
| N2 supplement | Invitrogen | 17502-048 |
| SB-431542 | Sigma Aldrich | S4317 |
| LDN-193189 | Sigma Aldrich | SML0559 |
| DMEM/F12 | Invitrogen | 31330038 |
| B27 supplement | Invitrogen | 17504-044 |
| B27 Plus supplement | Gibco | A35828-01 |
| Creatin monohydrate | Sigma Aldrich | C3630 |
| Interferon-γ | Peprotech | 300-02 |
| Neurotrophin-3 | Peprotech | 450-03 |
| Ascorbic acid | Sigma Aldrich | A5960 |
| db-cAMP | Sigma Aldrich | D0260 |
| Neurobasal Electro Medium | Thermo Fisher | A1413701 |
| B-27 Electrophysiology supplement | Thermo Fisher | A1413701 |
| Glutamax | Thermo Fisher | A1286001 |
| human recombinant GDNF | RnD Systems | 212-GMP-010 |
| human recombinant BDNF | Peprotech | 450-02 |
| Triiodothyronine (T3) | Merck | T2877 |
| Gentle Cell Dissociation Reagent | Stemcell Technologies | 100-0485 |
| Polyethyleneimine (PEI) | Sigma-Aldrich | 181978 |
| Accutase | Stemcell Technologies | 07920 |
| Dimethyl sulfoxide (DMSO) | Carl-Roth | A994.1 |
| Anti-adherence rinsing solution | Stemcell Technologies | 07010 |
| Matrigel | Corning | 354277 |
| Tubastatin A | Sigma Aldrich | SML004 |
| Suberoylanilide hydroxamic acid (SAHA) | Biomol | Cay10009929 |
| Poly-d-lysine (PDL) | Merck | P0899 |
| Chloroquine | Sigma | C6628 |
| Poly-HEMA | Merck | P3932 |
| Paraformaldehyde | Sigma Aldrich | P6148 |
| Goat serum | Sigma Aldrich | G9023 |
| Triton X-100 | Sigma Aldrich | T8787 |
| Poly-L ornithine | Sigma Aldrich | P3655 |
| Tricarballylic acid | Sigma Aldrich | T53503 |
| Methanol | Roth | N41.1 |
| Chloroform | Roth | 3313.1 |
| Natriumchloride | Roth | 3957.1 |
| Methoxyamine hydrochloride | Thermo Fisher | 10440364 |
| Ribitol | Sigma Aldrich | A5502-5G |
| Aqua-Poly/Mount | Polysciences Inc. | 18606-20 |
| Critical commercial assays | | |
| Cell Titer-Blue® Viability Assay (CTB) | Promega | G8080 |
| Lactate dehydrogenase Assay (LDH) | Promega | G7890 |
| Cell Proliferation BrdU Assay | Sigma Aldrich | 11669915001 |
| RNeasy Mini Kit | Qiagen | 74104 |
| QuantiTec Reverse Transcription Kit | Qiagen | 205314 |
| Quanti Fast SYBR Green Kit | Qiagen | 204057 |
| Deposited data | | |
| RNA Sequencing |  | GEO: GSE240972 |
| Experimental models: Cell lines | | |
| hiPSC-IMR90 clone 4 (IMR90^WT^) | WiCell | RRID:CVCL_C437 |
| CS789 | Prof. Dr. Egly, IGBMC Strasbourg | N/A |
| CS789^Res^ | GEMD, Leibniz IUF Düsseldorf | N/A |
| IMR90^KO^ | GEMD, Leibniz IUF Düsseldorf | N/A |
| Oligonucleotides | | |
| Primers listed in supplemental information table S2. | Eurofins | N/A |
| Recombinant DNA | | |
| PX458 | Addgene | 48138 |
| Software and algorithms | | |
| CRISPR design tool CHOPCHOP | https://chopchop.cbu.uib.no/ | N/A |
| CRISPRnano | Nguyen et al. (2022). 10.1093/nar/gkac440^73^ | N/A |
| Fiji Image J (v1.53f51) | https://imagej.net/software/fiji/ | N/A |
| Image Studio Lite (v5.2) | LI-COR Biosciences | N/A |
| Axion Integrated Studios (AxIS) navigator software (version 3.1.2) | Axion Biosystems | N/A |
| Neural Metric Tool software (version 3.1.7) | Axion Biosystems | N/A |
| MassHunter Qualitative software (vb08) | Agilent Technologies | N/A |
| NIST14 Mass Spectral Library | https://www.nist.gov/srd/nist-standard-reference-database-1a-v14 | N/A |
| GraphPad Prism (v9.5.1) | https://www.graphpad.com | N/A |

**SI Table S4. Neurotransmitter Metabolomics, raw data – Related to ‘Altered GABA levels and KCC2 expression hint towards a delayed GABA switch in disease cell lines’**.

See attached Excel sheet.

**References**

1. Laugel, V., Dalloz, C., Durand, M., Sauvanaud, F., Kristensen, U., Vincent, M.C., Pasquier, L., Odent, S., Cormier-Daire, V., Gener, B., et al. (2010). Mutation update for the CSB/ERCC6 and CSA/ERCC8 genes involved in Cockayne syndrome. Hum. Mutat. *31*, 113–126. 10.1002/humu.21154.
